# The Role of Neuroglia in the Development and Progression of Schizophrenia

**DOI:** 10.3390/biom15010010

**Published:** 2024-12-25

**Authors:** Neha S. Rawani, Allen W. Chan, Kathryn G. Todd, Glen B. Baker, Serdar M. Dursun

**Affiliations:** Neurochemical Research Unit and Bebensee Schizophrenia Research Unit, Department of Psychiatry and Neuroscience and Mental Health Institute, University of Alberta, Edmonton, AB T6G 2G3, Canada; nrawani@ualberta.ca (N.S.R.); awchan@ualberta.ca (A.W.C.); kathryn.todd@ualberta.ca (K.G.T.); dursun@ualberta.ca (S.M.D.)

**Keywords:** neuroglia, microglia, astrocytes, oligodendrocytes, transmitters, antipsychotics, neuro-inflammation, oxidative stress, myelination, blood–brain barrier

## Abstract

Schizophrenia is a complex heterogenous disorder thought to be caused by interactions between genetic and environmental factors. The theories developed to explain the etiology of schizophrenia have focused largely on the dysfunction of neurotransmitters such as dopamine, serotonin and glutamate with their receptors, although research in the past several decades has indicated strongly that other factors are also involved and that the role of neuroglial cells in psychotic disorders including schizophrenia should be given more attention. Although glia were originally thought to be present in the brain only to support neurons in a physical, metabolic and nutritional capacity, it has become apparent that these cells have a variety of important physiological roles and that abnormalities in their function may make significant contributions to the symptoms of schizophrenia. In the present paper, we review the interactions of brain microglia, astrocytes and oligodendroglia with aspects such as transmitter dysregulation, neuro-inflammation, oxidative stress, synaptic function, the gut microbiome, myelination and the blood–brain barrier that appear to affect the cause, development and treatment of schizophrenia. We also review crosstalk between microglia, astrocytes and oligodendrocytes and the effects of antipsychotics on neuroglia. Problems associated with studies on specific biomarkers for glia in schizophrenia are discussed.

## 1. Introduction

Schizophrenia is a complex and heterogenous psychiatric disorder [1,2,3] characterized by the presence of constellations of positive (hallucinations, delusions, disorganized thinking and behaviour) and negative (blunted affect, alogia, avolition, asociality) symptoms as well as cognitive impairment [4,5,6]. The phases of the disorder include the prodromal (propsychotic) phase, initial onset of psychosis and chronic illness [7,8]. Schizophrenia is considered to be a neurodevelopmental disorder involving interactions among multiple genetic, epigenetic and environmental factors [6,9,10,11,12,13,14,15,16,17]. This disorder has a worldwide prevalence of approximately 0.7–1% [16,17].

Although the emphasis of studies on the etiology and development of drugs for the treatment of schizophrenia focused for many years on the dysregulation of neurotransmitters [primarily dopamine, but also glutamate, serotonin and γ-aminobutyric acid (GABA)] [18,19,20,21,22,23,24,25,26,27], it became obvious that factors in addition to these neurotransmitters were involved. Studies in recent years have also focused on other neurotransmitters and neuromodulators (e.g., acetylcholine, D-serine) and other factors such as the immune, endocrine and endocannabinoid systems, oxidative stress, mitochondrial dysfunction, the blood–brain barrier (BBB) and the gut–brain axis [2,11,27,28,29,30,31,32,33,34,35,36,37,38,39,40,41,42,43,44,45,46,47,48,49].

There is growing evidence that glial cells (also known as neuroglia), which affect many of the aspects mentioned above, should also be considered important factors in schizophrenia. The principal types of glia in the central nervous system (CNS) are microglia, astrocytes, oligodendrocytes, ependymal cells, radial glia and neuron-glia antigen 2 (NG2) cells (also called oligodendrocyte precursor cells or polydendrocytes), while in the peripheral nervous system (PNS) the principal types are Schwann cells, satellite cells and enteric glia [50,51,52]. Although glia were originally thought to be important in brain function only because of their physical, metabolic and nutritional supportive role for neurons, it has become obvious in recent decades from genetic and molecular evidence that this approach was too “neurocentric” [51,53] and that glia have many roles that may be important in the normal functioning of the brain but under pathological conditions can contribute to the symptoms of neuropsychiatric disorders [2,8,53,54,55,56,57,58,59,60,61]. The aim of the present paper is to provide a review of the proposed roles of microglia, astrocytes and oligodendrocytes in normal brain function and in the etiology and pharmacotherapy of schizophrenia.

## 2. Microglia

Microglia are the resident immune cells of the CNS. Associated with their innate immune function, they express a number of pathogen recognition receptors including Toll-like receptors and scavenger receptors [62]. Microglia are involved in coordinating neuro-inflammatory responses in the CNS, but, as mentioned below, make other contributions to normal brain function, including synaptic pruning [63]. However, in pathological situations including schizophrenia, microglia may become chronically active, creating an inflammatory state. This is of great interest since there is now a large body of evidence suggesting immune system abnormalities and increased neuro-inflammation in a substantial number of patients with schizophrenia [64,65,66,67,68,69,70,71,72,73,74,75,76,77,78,79,80,81,82,83,84] and the anti-inflammatory properties of several antipsychotics [85].

Microglia can be activated by diverse factors including cytokines, cellular debris and bacterial lipopolysaccharide (LPS) which may be released by various stress events [6]. In their activated state, there can be two subtypes, the classical M1 microglia that release inflammatory mediators and alternative M2 microglia that release anti-inflammatory mediators and can produce neuroprotective effects [86,87]. There is also a pool of different subtypes of microglia with a diversity of expressions of receptors and morphology [87]. Activated M1 microglia release free radicals and pro-inflammatory cytokines such as interleukin-6 (IL-6), IL-8, IL-1β and tumour necrosis factor-α (TNF-α) [37] which reciprocally influence and modulate neuronal function [88,89].

Microglia express, and can de novo express or upregulate, receptors for several neurotransmitters and neuromodulators, such as glutamate, adenosine triphosphate (ATP), GABA, dopamine, noradrenaline, serotonin and endocannabinoids [53,62,90], and activation of these receptors can regulate the release of a number of microglia effectors such as IL-1β, brain-derived neurotrophic factor (BDNF) and nitric oxide [53,91]. For example, there is a large body of evidence implicating the hypofunction of N-methyl-D-aspartate glutamate receptors (NMDARs) and the subsequent increased release of glutamate in the etiology of schizophrenia [23,24,25], and glutamate has also been proposed to interact with microglial cells to stimulate the over-production of pro-inflammatory cytokines, neuro-inflammation, dendritic apoptosis and excessive synaptic pruning in psychosis [56,92,93,94,95]. The promotion of neurogenesis and synaptogenesis by secretion of neurotrophic factors is reported to be a normal function of microglia [87]. BDNF, a neurotrophic factor released by microglia and reported to enhance learning-related synapse formation, particularly glutamatergic synapses [96,97], has been reported to be lower than control values in plasma samples from patients with schizophrenia [98].

Increased microglial activation and neuro-inflammation which is positively correlated with psychotic severity has been reported in people with an ultra-high risk of developing psychosis [94]. It appears that the activation of microglia and elevation of levels of pro-inflammatory cytokines in schizophrenia occur prior to the onset of psychotic symptoms, suggesting that inflammatory status may predict the subsequent onset of psychotic symptoms [94,95]. Chronic microglial activation has been reported to be associated with cytokine release and inflammation, excessive synaptic pruning, reduced cortical volume in the brain and prefrontal cortex (PFC) dysfunction [37]. However, the literature on inflammation in schizophrenia and the involvement of microglia activation in inflammation is not without controversy [59,68,99,100,101,102,103,104]. In a genome-wide association study (GWAS) on data from a large cohort of people with schizophrenia and healthy controls, Goudriaan et al. [100] found that astrocyte and oligodendrocyte gene sets were associated with increased risk for schizophrenia, but microglia gene sets were not. They also reported that the astrocyte and oligodendrocyte results were related to astrocyte signaling at the synapse, the integrity of myelin-containing membranes, the development of glia and epigenetic control. Trepanier et al. [68] conducted a systematic review of the literature on neuro-inflammatory marker studies on the postmortem brain tissue of patients with schizophrenia. The expression of glial fibrillary acidic protein (GFAP), a marker for astrocytes, was elevated, lower or not changed compared to controls in 6, 6 and 21 studies, respectively, while microglial markers were elevated, lower or unchanged in 11, 3 and 8 studies, respectively. Snijders et al. [101] conducted a meta-analysis of studies on microglia in schizophrenia and also conducted their own investigation by performing immunostaining and qPCRs on an additional dataset. They concluded that the expression of several microglia-specific genes was decreased in schizophrenia and proposed that there was a change in microglial phenotype rather than density in schizophrenia. It has been proposed [102] that in patients with schizophrenia, infiltrated regulatory T lymphocytes activate astrocytes, which then increase transforming growth factor-β (TGFβ) secretion, forcing microglia to be sustained in a non-inflammatory state, promoting microglial phagocytosis and synaptic pruning [102]. Murphy and Weickert [103] have provided an interesting commentary on the controversies related to microglia and neuro-inflammation in schizophrenia. Koskuvi et al. [104] studied human-induced pluripotent stem cell (hiPSC)-derived microglia generated from monozygotic twins discordant for schizophrenia, and from healthy controls. These cells were used to study the transcriptional and functional differences in microglia between the affected and unaffected twins and the controls. Although there was an increased expression of inflammatory genes in the cells from the twins with schizophrenia, there were not clear functional signs of hyperactivation in microglia in those cells. The major histocompatibility complex (MHC) locus has a strong association with schizophrenia, and microglia are major MHC class II-expressing cells in the brain; these researchers found an upregulation of these genes only in the microglia from the affected twins [104].

Through interactions with the innate and adaptive immune systems, the complement system of plasma and membrane proteins is involved in modulating tissue homeostasis and in immune surveillance [105]. The complement cascade is involved in clearing debris, enhancing inflammation and tagging pathogens for engulfment or destruction, and is an important contributor to synapse elimination and plasticity [106,107]. Neurons, microglia and astrocytes can produce complement compounds [106,107]). The appropriate activation of the complement system plays an important role in the normal functioning of the brain, but overactivation or dysregulation may lead to synaptic dysfunction and an inflammatory response that is excessive [107]. Activation of the complement system can trigger microglia-dependent synaptic elimination through complement receptor 3 (CR3) [106], but there is also evidence of increased synaptic loss, increased microglia synaptic engulfment and excess pruning due to the overexpression of C4, a gene involved with inflammatory responses and associated with schizophrenia [106,108,109].

There are also reports of bidirectional interactions between microglia and mitochondrial dysfunction in schizophrenia. Reactive oxygen species (ROS) produced by mitochondria may lead to the increased production of pro-inflammatory cytokines by microglia [110], and the mitochondrial anti-oxidative defence system may be perturbed by increased pro-inflammatory cytokines in patients with schizophrenia [77,111]. In a study of postmortem PFC tissue from patients with chronic attack-like progressive schizophrenia or continuous schizophrenia, Vikhreva and Uranova [112] concluded that the former involves the increased reactivity of microglia at a young age and dystrophic changes in microglia that increase with age and length of disease, while the latter is associated with the decreased reactivity of microglia and non-progressive dysphoric changes.

It has been postulated for many years that early inflammation may be a contributing factor to schizophrenia, and studies in maternal immune activation (MIA) models in rodents have been used to demonstrate that a maternal infection can result in immunological changes that mobilize microglia and may result in psychosis-like symptoms in the offspring [37,63,95,113,114]. Changes in several neurotransmitter systems, including the dopamine and glutamate systems, have been found in such models [37,95]. The microglial two-hit model of schizophrenia [114] proposes that the perinatal activation of microglia puts them in a primed state, and that later stress in adolescence can trigger their overactivation and the excessive pruning of synapses in brain areas such as the PFC and hippocampus [6].

Animal models of schizophrenia based on administration of the NMDAR antagonists phencyclidine (PCP), ketamine or dizocilpine (MK-801) have been reported to produce a state of neuro-inflammation characterized by microglial reactivity and the excessive production of pro-inflammatory cytokines [114].

It has also been proposed that there is a disruption of the BBB in some patients with schizophrenia [115,116] and that activated microglia, through the secretion of ROS and pro-inflammatory cytokines, can also cause the disruption of the BBB [31].

The contributions of the gut microbiome to glial function in schizophrenia must also be considered. There is now a very large body of evidence indicating the influence of the gut microbiome on brain function [117,118,119]. The activity of the gut microbiome can have marked effects on the immune system and inflammation, neurotransmission, the hypothalamic–pituitary–adrenal (HPA) axis, myelination and the BBB [31,117,118,119,120,121,122]. Preclinical and clinical studies provide evidence for dysregulation in the gut microbiome in schizophrenia-spectrum disorders [123,124,125,126,127,128,129]. It has been reported that the gut microbiome is involved in the regulation and function of microglia [118,130]. Ju et al. [131] have reported that short-chain fatty acids (SCFAs) produced by gut microbes cross the BBB and modulate the activity of microglia and the production of cytokines.

## 3. Astrocytes

Astrocytes are normally the most abundant glial cells in the CNS. These cells are associated very closely with neurons; cell bodies and synapses (tripartite synapses) enwrapped by astrocytes are involved in the uptake and release of transmitters, particularly glutamate, by the neuron, and also in the production and release of modulatory factors [132,133]. It has been proposed that astrocytes play an important role in synaptogenesis and that the temporal relationship between the maturation of astrocytes and synapses suggests that bidirectional interactions between them are involved in the post-natal maturation of both, resulting in the fine-tuning of the development of functional circuits [132,134,135,136]. See [132] for detailed tables of the signal-related molecules and synaptogenesis-related molecules secreted by astrocytes. When exposed to damaging factors secreted by activated neuro-inflammatory microglia, astrocytes can be activated and, like microglia, show a dual nature [86]. A1 astrocytes release IL-1β, TNF-α and C3 components (which propagate neuro-inflammation), D-serine and nitric oxide, while A2 astrocytes release anti-inflammatory compounds such as neurotrophic factors and anti-inflammatory cytokines and promote the survival, growth and repair of neurons [86]. BDNF secreted by astrocytes has been reported to be involved in the modulation of GABAergic synapses [137]. BDNF and ciliary neurotrophic factor (CNTF) contribute to the development and survival of oligodendrocytes [138], and it has been reported that following white matter damage, BDNF secreted by astrocytes promotes oligodendrogenesis [139].

In a recent paper, Ling et al. [140] used single-nucleus RNA sequencing to analyze postmortem prefrontal cortical tissue from 97 healthy controls and 94 people with schizophrenia. They described a neuron–astrocyte relationship in which samples from people whose neurons strongly expressed genes encoding synaptic components also showed astrocytes that more strongly expressed genes with synaptic functions and genes for the synthesis of cholesterol, an important component of synaptic membranes. They termed this the synaptic neuron and astrocyte program (SNAP) and reported that this concerted program declined in ageing and schizophrenia [140]. Zehnder et al. [136] reported that the development of mitochondrial biogenesis in astrocytes is important in regulating astrocyte maturation and promoting synaptogenesis. These authors surmised that astrocytic mitochondria may be a potential therapeutic target in treating disorders such as schizophrenia in which there is impaired synaptogenesis. Mounting evidence suggests that astrocytes are also involved in the regulation of rhythmic activity and the synchronization of neuronal networks [141].

**Figure 1:**
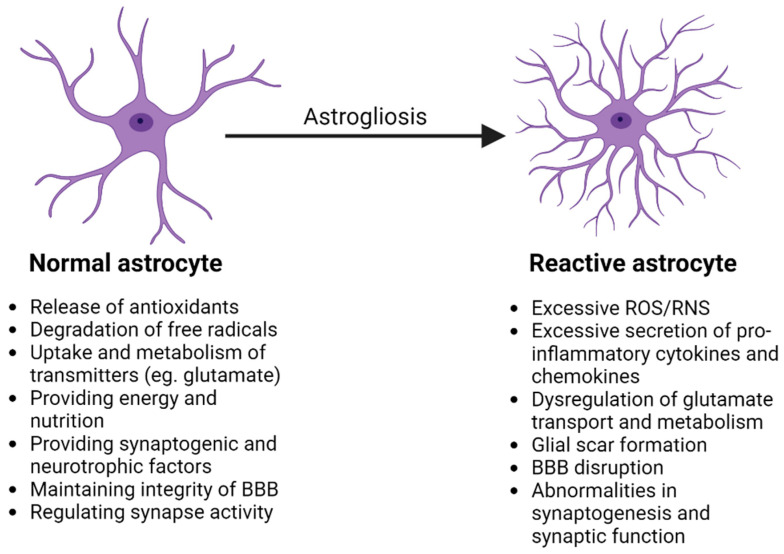
Examples of properties of normal astrocytes and reactive A1 astrocytes which have been reported to be relevant to normal brain function and brain function in schizophrenia, respectively (adapted from [146]). Astrogliosis may be increased by factors arising from microglia such as C1q, pro-inflammatory cytokines and free radicals [146]. This figure is based on information obtained from references cited in the text of this review [2,12,41,54,60,133,141,146]. Created in BioRender, Chan, A. (2025) https://BioRender.com/e61n459, accessed on 20 September 2024.

Astrocytes express numerous types of receptors, transporters, enzymes and ion channels [2]. These neuroglia cells contribute to homeostasis in the CNS through a number of mechanisms, including the following: providing nutrition to neurons; regulating the uptake and release of neurotransmitters, particularly glutamate; controlling ion and water availability; maintaining redox balance; regulating synapse formation and function; modulating cerebral blood flow and metabolism; contributing to the proper development and functioning of the BBB; regulating iron transport; and providing defense against oxidative stress [2,8,41,114,142,143,144,145,146,147]. It has been proposed that dysfunction related to the maturation of astrocytes may result in abnormalities in mitochondrial biogenesis, synaptogenesis and the glutamatergic and dopaminergic transmission characteristics of schizophrenia [41]. For a schematic overview of the properties of astrocytes proposed to be involved in normal brain function and in schizophrenia, see Figure 1.

Astrocytes are involved in the transfer of glucose and lactate to neurons [133], and in a study on the knockout of glutamate receptors, astrocytes have been reported to provide the majority of functional glutamate transport [148]. GABA-A and -B receptors and GAT-1 and -3 transporters for GABA are also expressed by astrocytes [149]. By secreting synapotogenic and neurotrophic factors including thrombospondins, hevin, and TGF-β1, astrocytes can modulate synaptic function [149]. In contrast, astrocytes are also capable of eliminating synapses by mechanisms which include direct phagocytosis, the stimulation of microglia to phagocytose, and the activation of the intracellular inositol 1,4,5-triphosphate (IP_3_) pathway, producing release of Ca^2+^ from the endoplasmic reticulum [2].

**Figure 2:**
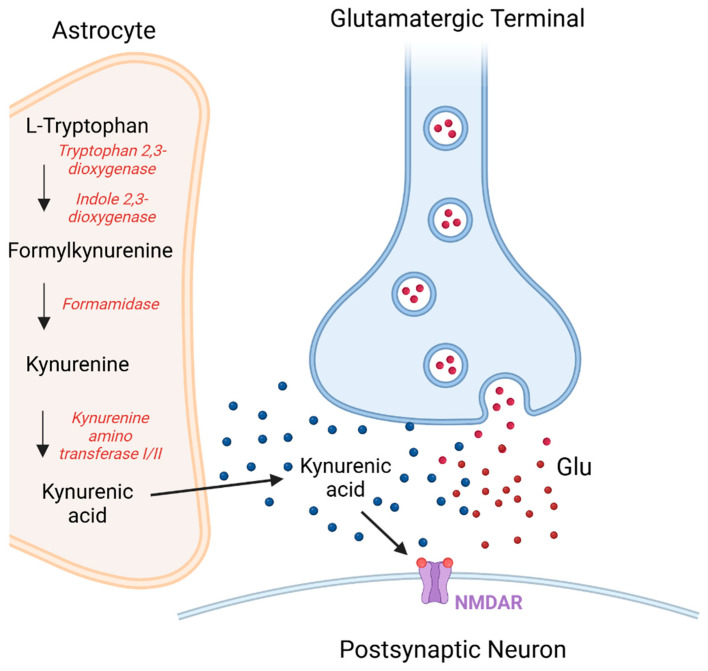
The possible contribution of increased kynurenic acid to the NMDA glutamate receptor hypofunction proposed in schizophrenia. The kynurenine pathway is a major route for the metabolism of tryptophan, and in the brain, kynurenine is metabolized to kynurenic acid in astrocytes as part of that pathway. The released kynurenic acid acts as an antagonist at the glycine co-agonist site on the NMDAR [27,63,156,157]. The above figure is adapted from information from references [27,63,132,158]. Glu = Glutamate. Created in BioRender, Chan, A. (2025) https://BioRender.com/e72n912, accessed on 28 November 2024.

Astrocytes also make an important contribution to the glutamate–glutamine cycle. Glutamate released from neurons is transported into astrocytes, and there it is metabolized to glutamine. The glutamine is subsequently transported to neurons and converted to glutamate [8,150]. Elevated glutamine-to-glutamate ratios have been reported in the CSF of patients with first-episode psychosis (FEP) or drug-naïve schizophrenia [151,152]. Bernstein et al. [153] reported a reduced density of astrocytes expressing the disrupted-in-schizophrenia 1 (DISC1) gene in the dentate gyrus in patients with schizophrenia; such a change could result in the reduced synthesis of D-serine [153], a potent NMDAR co-agonist that has been implicated in the etiology and treatment of schizophrenia [154,155]. Decreases in D-serine levels and increases in levels of kynurenic acid, a tryptophan metabolite and NMDAR antagonist derived from astrocytes, could contribute to the hypofunction of NMDARs in schizophrenia [27,37,63] (Figure 2).

The association of D-serine with astrocytes has been a matter of controversy [159,160], with early research proposing that it is synthesized in astrocytes [159], but later studies indicating that L-serine is present in astrocytes and that it is shuttled to neurons to be converted to D-serine, and that neuronally based D-serine regulates NMDAR activity [160,161]. However, it has also been proposed that astroglia, by removing synaptic D-serine and by regulating its subsequent metabolic degradation, can influence NMDAR activity [162]. Although kynurenic acid acts as an antagonist at the three ionotropic glutamate receptors, it preferentially inhibits the glycine co-agonist site on the NMDAR [163]. It has also been proposed as a non-competitive allosteric inhibitor of the α-7 nicotinic receptor [164], although there has been some controversy in that regard [165,166,167]. In the brain, kynurenic acid is formed in part of the kynurenine pathway of tryptophan metabolism [27,63,132,158]. It has been reported to modulate glutamatergic, GABAergic, cholinergic and dopaminergic transmission [reviews: 132,168]. Kynurenic acid has been proposed to be neuroprotective in some neurological disorders [168,169], but its aberrant production has been reported to be connected to schizophrenia pathology [132,163,170,171,172]. There have been numerous reports of elevated levels of kynurenic acid in the CSF and postmortem brain tissue of patients with schizophrenia, but results in plasma have not been consistent [163,172].

Changes in astrocytic density and/or several astrocytic markers have been reported in postmortem studies of schizophrenia, although the reports have not been consistent [2,59,82,173,174]. Laricchuita et al. [59] conducted a systematic review of the glial hallmarks of schizophrenia, with a focus on astrocytes and microglia, and concluded that the development of schizophrenia may involve changes in the density, morphology and function of these two types of glia. These researchers also discussed conflicting reports in the literature on the overactivation of astrocytes and microglia in various brain regions in schizophrenia. They also provided an overview of confounding factors that should be considered in studies of biomarkers for astrocytes and microglia. Pinjari et al. [175] measured plasma levels of S100B, P-selectin (a cellular adhesion molecule) and IL-6 in patients with schizophrenia and healthy controls. Levels of P-selectin correlated positively with levels of S100B and IL-6, and it was postulated that in patients with schizophrenia, peripheral immune activation may be related to neuro-inflammation and the activation of astrocytes. Myo-inositol is highly expressed in astrocytes, and low levels may be an indicator of the impaired activity of astrocytes, possible redox imbalance, excitotoxicity and inappropriate astrocyte-mediated inflammatory defence [59]. Jeon et al. [176] measured myo-inositol levels (using 7-Tesla magnetic resonance spectroscopy) in the anterior cingulate cortex in healthy controls and in patients with FEP schizophrenia (at baseline and after 6 months of treatment with antipsychotics). At baseline, levels of myo-inositol were lower in the patients with schizophrenia than in the healthy controls, while there was no difference between the groups after months of treatment of the patients with antipsychotics. The complement system should also be considered in the actions of astrocytes. In a study on human postmortem brain tissue, Mou et al. [109] showed the robust expression of C4 in the subventricular zone (SVZ), a region critical to brain development, and that this expression was increased in patients with schizophrenia relative to controls.

Oxidative stress and neuro-inflammation can also be influenced by astrocytes [146]. Normally, astrocytes have antioxidant responses via the production of antioxidants (e.g., glutathione), removal of glutamate and stimulation of antioxidant systems such as nuclear factor erythroid 2-related factor 2 (Nrf2). However, in pathological situations such as the dysfunction of mitochondria, impairment of metabolism, excessive glutamate and/or reduced production of antioxidants, astrocytes can, through the release of ROS or reactive nitrogen species (RNS), produce the activation of microglia and neuro-inflammation [148]. Oxidative stress can also affect astrocytes adversely through effects on metabolism and the transport of glutamate by astrocytes [147,177].

As mentioned above, the development, maintenance and function of the BBB can be affected by astrocytes. There is now evidence of BBB dysfunction in some patients with schizophrenia [12,31,115]. Disruption of the BBB secondary to neuro-inflammation has been proposed, and subsequent leakage of the BBB may permit an increased infiltration of pro-inflammatory factors [116].

The BBB endothelium functions within a modular neurovascular unit composed of a capillary segment and its affiliated pericytes, basement membranes, astrocytes, microglia and neurons [12,31]. Alterations in this unit such as changes in ion channel and drug transporter expression on endothelial cells and glia, leakage at tight junctions, and abnormal modulation of adhesion molecules and leucocytes may occur in schizophrenia [12,31]. Pollak et al. [31] have provided extensive details on the abnormalities of BBB-associated molecules in psychotic disorders and the effects of risk factors for these disorders on BBB function. Although there is controversy about the validity of S100B, an acidic calcium-binding protein secreted by astrocytes and oligodendrocytes, as a marker of BBB disruption [178], there have been reports of higher serum and CSF S100B concentrations in patients with schizophrenia compared to controls [179,180]. In a meta-regression analysis, Schumberg et al. [180] found that serum S100B levels were higher in patients with schizophrenia than in controls and were related to the duration of illness and clinical symptomology.

Interestingly, astrocytes can have a dual effect on the BBB since several vascular permeability factors derived from astrocytes can worsen BBB disruption, while protective factors from these glia can reduce the increase in BBB permeability [181].

## 4. Oligodendrocytes

Oligodendrocytes are the myelinating cells of the CNS, and thus play a crucial role in the propagation of action potentials and neuronal communication. They have other functions as well, including providing trophic actions, energy supplies and buffering [63]. Oligodendrocytes are also thought to have the ability to downregulate inflammatory damage [182,183].

In recent years, a number of researchers have provided evidence supporting abnormalities in myelination in some cases of schizophrenia. The abnormal myelination of connecting fibres in the left frontotemporal region, an area of the brain proposed to be involved in the development of auditory and verbal hallucinations, has been reported in patients with schizophrenia [184]. Discrepancies in sensory feedback mechanisms may be caused by dysmyelination-related delays in patients with psychosis [185]. Zhang et al. [186] reported that mice treated with the NMDAR antagonist phencyclidine *(*PCP*)* displayed schizophrenia-like behaviours, impaired myelination in the frontal cortex and decreased quantities of oligodendrocytes. Schizophrenia-like behavioural deficits have also been reported in myelin gene knockout mice [185]. Since some patients with myelin-related disorders have also exhibited psychosis, it has been postulated that interruptions of myelination in localized regions such as the frontotemporal, callosal and periventricular fibre tracts contribute to psychotic behaviour [187].

Reductions in the number and density of oligodendrocytes in the postmortem brain tissue of patients with schizophrenia have been reported [185,187]. Other postmortem studies on brain tissue from patients with schizophrenia reported a decrease in the volume and mitochondrial number of oligodendrocytes in the caudate nucleus and prefrontal areas, and decreased numbers of oligodendrocytes and myelin volume in the anterior thalamus nucleus [188]. Oligodendrocyte precursor cells (OPCs) have been reported to be very sensitive to oxidative stress [189]. Maas et al. [190] have proposed that environmental, genetic and epigenetic factors combine to lead to the accumulation of ROS in these precursor cells, disrupting signal transduction processes and resulting in hypomyelination and disrupted connectivity in the PFC in schizophrenia. Windrem et al. [191] produced a humanized chimeric mouse model via engrafting glial progenitor cells [from induced pluripotent stem cells (iPSCs) obtained from patients with juvenile-onset schizophrenia and age-matched controls] into neonatal, congenitally hypomyelinated mice. The chimera derived from the patients with schizophrenia exhibited impaired maturation of oligodendrocytes and astrocytes and a number of behavioural deficits characteristic of schizophrenia [191].

## 5. Crosstalk Among Neuroglia

**Figure 3:**
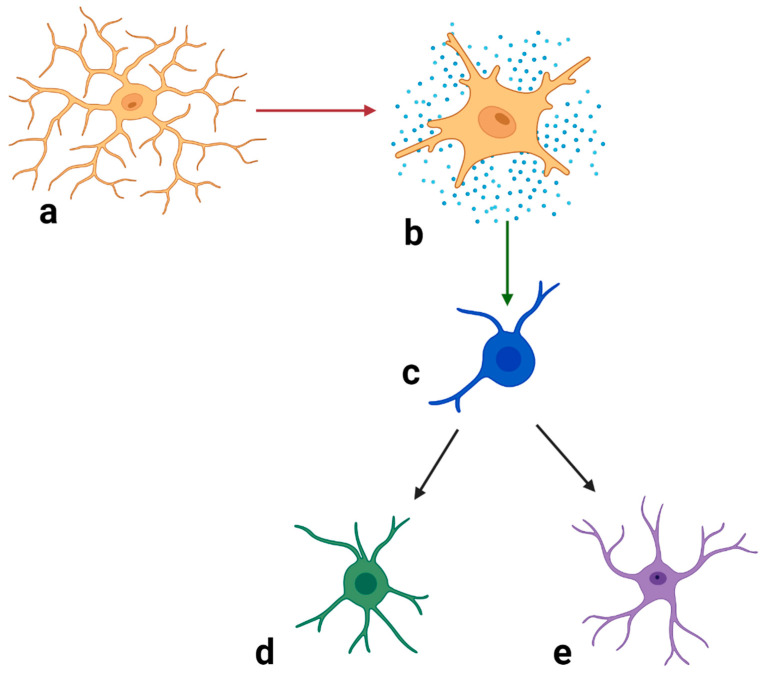
Interactions of neuroglia which may be relevant to schizophrenia. Figure adapted from Deitz et al. [8]. It is proposed that immune activation of microglia and release of pro-inflammatory factors such as cytokines during fetal development leads to suppression of differentiation of glial progenitor cells and resultant production of immature oligodendrocytes and astrocytes with reduced function. Immature oligodendrocytes result in hypomyelination, reduced white matter integrity and circuit dysfunction. Immature astrocytes lead to abnormalities in the following: glutamate transport, potassium buffering, release of neurotrophic factors and synaptic function [8,63,176]. (**a**) = microglial cell; (**b**) = activated microglial cell; (**c**) = glial progenitor cell; (**d**) = immature oligodendrocyte; (**e**) = immature astrocyte. Created in BioRender, Chan, A. (2025). https://BioRender.com/j61o876, accessed on 20 September 2024.

Crosstalk among microglia, astrocytes and oligodendrocytes may also be relevant with regard to schizophrenia. Microglia and astrocytes can influence the differentiation of OPCs into myelinating oligodendrocytes, thus affecting remyelination [192,193].Domingues et al. [192] have provided a comprehensive review describing astrocyte-derived promoters and inhibitors of oligodendrocyte proliferation, differentiation and myelination and the importance of astrocyte activation in these interactions. These authors also describe how microglia can affect OPC survival and differentiation and modulate remyelination and demyelination through the release of ROS and RNS, glutamate, neurotrophic factors and cytokines or chemokines, and how astrocytes and the subsequent recruitment of microglia act through the clearance of myelin debris during myelination [192]. Microglia are present in the CNS prior to the onset of neurogenesis and guide neurogenesis and astrogliogenesis [193]. Although astrocytes normally promote the survival of neurons and synaptogenesis, when they are activated by the secretion of complement component 1q (C1q) and cytokines by activated microglia, astrocytes lose the above-mentioned positive effects and may induce the death of neurons and oligodendrocytes [194]. Dietz et al. [8] have proposed that in schizophrenia the activation of microglia during embryogenesis results in the delayed differentiation of astrocytes and oligodendrocytes, leading to abnormalities in cortical and subcortical white matter integrity. See Figure 3 for a schematic representation of this process. It has been proposed that activated microglia, through the release of nitric oxide, peroxynitrite and inflammatory cytokines, may produce cytotoxicity in oligodendrocytes [195,196,197]. The loss of oligodendrocytes could not only result in impaired myelination but also in increased infiltration by pro-inflammatory cytokines and microglia [198].

Uranova et al. [198], using transmission electron microscopy and morphometry, conducted a study on microglia and adjacent oligodendrocytes in postmortem PFC tissue extracted from patients with schizophrenia exhibiting predominantly positive or negative symptoms and from healthy controls. They found activation of microglia and dysphoric alterations of both microglia and oligodendrocytes in close proximity to each other in both schizophrenia groups compared to controls. In both clinical groups there was a reduction in volume density and the number of mitochondria and an increase in the number of lipofuscin granules in both glia types, and it was suggested that microglial dystrophy may be contributing to oligodendrocyte dystrophy in the patients, particularly those with positive symptoms during relapse.

## 6. Interactions of Antipsychotics with Neuroglia

Although the focus on the mechanisms of antipsychotics has been primarily on interactions with neurotransmitter receptors, there is now a relatively large body of evidence indicating that many of these drugs have important effects on glia. Konopeske et al. [199] reported that the chronic administration of antipsychotics caused a significant reduction in the numbers of astrocytes in macaque monkeys. Several antipsychotics have been reported to reduce microglial activation and hence the release of pro-inflammatory cytokines [72,85,200,201,202,203,204]. Long et al. [205], in a study in which microglial cells were activated by LPS and then treated with haloperidol or risperidone, reported that risperidone produced stronger anti-inflammatory and neuroprotective actions than haloperidol and that these effects were mediated through p38 mitogen-activated protein kinases (MAPKS) and Janus kinase-signal transducer and activator of transcription (JAK-STAT) signaling pathways. These researchers proposed that these effects could contribute to the actions of risperidone on the negative symptoms of schizophrenia. The number of more mature oligodendrocytes arising from OPCs was reported to be increased in the presence of haloperidol [206]. Akkouh et al. [207], in a study on human iPSC astrocytes, reported that clozapine induced the release of gliotransmitters (D-serine and L-glutamate) in clozapine-responsive cells, but not in those cells that were clozapine-resistant. In a recent magnetic resonance spectroscopy (MRS) study on patients with schizophrenia and healthy controls, Torres-Carmona et al. [208] reported higher levels of myo-inositol (a surrogate marker of astrocytic activity) in the patients who responded to clozapine than in those who did not in several brain areas related to schizophrenia neurobiology. Yuhas et al. [209], in a study on human-derived astroglia (A172) cells, found that clozapine produced downregulation of the expression and release of TNFα, IL-β and IL-8 (pro-inflammatory cytokines), and upregulated the expression of cyclooxygenase2 (COX2). Clozapine has been reported to reduce glutamate uptake in astrocytes through a mechanism involving a reduction in the expression of the GLT-1 glutamate transporter [210]. Tanahashi et al. [211], in a study on astrocytes in hippocampus cultures, found that clozapine enhanced the release of D-serine. In a study with cultured astrocytes, Okada et al. [212] found that quetiapine increased D-serine release, enhanced signaling related to the cellular regulators extracellular signal-regulated kinase (Erk), protein kinase B (PKB, also called Akt) and 5′-adenosine monophosphate-activated protein kinase (AMPK) and increased cyclic AMP synthesis. Using the MK-801 mouse model of schizophrenia, Yu et al. [213] proposed that quetiapine may reduce oligodendrocyte apoptosis, promote oligodendrocyte-induced myelination through the modulation of PI3k/Akt signalling and thus reduce cognitive impairment in schizophrenia. However, it should be noted that it has also been reported that quetiapine does not reduce disease severity in a mouse model of autoimmune-mediated demyelination in the spinal cord [214]. In the neonatal ventral hippocampus lesion (nVHL) rat model of schizophrenia, Apam-Castillejos et al. [215] found that olanzapine reduced reactive astrogliosis as well as reducing inflammation and oxidative stress and improving neuronal plasticity in the PFC. Olanzapine has also been reported to stimulate the proliferation of oligodendrocytes [216]. The retinoid X receptor agonist bexarotene inhibits inflammatory responses, upregulates microglia phagocytosis [217], reduces the microglial release of pro-inflammatory cytokines, reduces numbers of A1 astrocytes [218] and has been reported in two clinical trials to be a useful adjunctive drug in the treatment of schizophrenia [219,220].

Interactions with glia can also account for some of the adverse effects observed with antipsychotics, particularly after chronic administration. Schmitz et al. [221] have provided a comprehensive table on the effects of several typical and atypical antipsychotics on multiple actions of glia (e.g., inhibition of the release of pro-inflammatory cytokines; effects on the uptake or release of glutamate and D-serine; release of trophic factors; oxidative stress) and have described in detail how some of these effects may contribute to the therapeutic actions of these drugs and how some may, particularly in association with long-term use and aging, contribute to undesired side effects (Janus face). These authors have suggested that the co-administration of glioprotective agents such as resveratrol could attenuate some of the side effects of these drugs and reduce glial reactivity [221]. Several antipsychotics have been reported in clinical and preclinical studies to cause brain volume loss and produce astrocyte death after chronic administration [review: 222]. In a recent study with a human astrocyte cell line (C1028), He et al. [222] found that chronic treatment with the antipsychotics olanzapine, quetiapine, risperidone and haloperidol induced the death of astrocytes and activated signaling modulated by the inflammasome sensor nod-like receptor protein3 (NLRP3) and caspase-1. These researchers reported that co-treatment with a histamine H1 receptor agonist reduced this activation and suggested that such drugs could be useful in the future development of strategies to inhibit the death of astrocytes caused by antipsychotics and to inform the development of new antipsychotics with reduced toxicity.

## 7. Discussion and Future Directions

As can be seen from the above literature review, the etiology and pharmacotherapy of schizophrenia are very complex, with many facets beyond just the dysregulation of a small number of neurotransmitters involved. It is also now evident that neuroglia have many important roles in brain function above and beyond just providing physical and metabolic support for neurons, and that their dysfunction may be very pertinent to the etiology of schizophrenia. These relevant roles include possible involvement with synaptic development and function, inflammation, endocrine function, oxidative stress, mitochondrial dysfunction, the BBB and the gut–brain axis. Several of the current antipsychotic drugs are now known to have effects on glia in addition to their effects on neurotransmitter receptors on neurons, and these actions may contribute to their therapeutic results as well as their side effects.

Research on glia has enhanced our understanding of the etiology of schizophrenia and the actions of currently available antipsychotics but has also emphasized how complex this disorder is. There are several impediments to doing future research on biomarkers and drug development for schizophrenia with regard to glia. As discussed in detail by Laricchiuta et al. [59], there are conflicting results on the overactivation of microglia and astrocytes and the involvement of inflammation in schizophrenia in the literature. In recent years, there has been an increased use of single-cell RNA-sequencing (scRNA-seq) or single-nucleus RNA sequencing (snRNA-seq) to study specific cell types in brain tissue from heathy controls and people with schizophrenia [223,224,225,226,227], and the findings of several studies do not support the major involvement of glia in schizophrenia. Skene et al. [223] studied whether the genomic loci proposed to be implicated in schizophrenia mapped onto specific cell types and reported consistent mapping to pyramidal cells, medium spiny neurons and certain interneurons but much less consistent mapping to embryonic, progenitor or glial cells. In a study on schizophrenia risk genes employing summary-data based Mendelian randomization based on single-cell sequencing data, Wu et al. [225] identified 54 new risk genes associated with schizophrenia. These researchers reported that the highest expression of schizophrenia risk genes was in excitatory neurons and caudal ganglionic eminence interneurons. Ruzicka et al. [226] used snRNA-seq to study postmortem PFC tissue from two cohorts of healthy people and people with schizophrenia and developed a single-cell resolution transcriptomic atlas of the PFC as well as characterizing the expression changes associated with schizophrenia. They included neuronal and glial cell types to investigate schizophrenia-dysregulated genes. Gene expression changes were observed in all detected cell types, but most changes occurred in neurons, with more than three-quarters of the changes occurring in excitatory neurons, with downregulation favoured. However, Thrupp et al. [227] concluded that snRNA-seq is not suitably sensitive for detecting cellular activation in microglia in humans.

Several of the possible biomarkers related to glia in schizophrenia are also present in other neuropsychiatric disorders. In addition, many of the receptors expressed by glia are also expressed by neurons. The clinical heterogeneity of schizophrenia, disagreement on diagnosis and the presence of at least three phases in this disorder are complicating aspects with regard to examining biomarkers [228,229], and measurement of biomarkers should be conducted over the clinical course of the disorder. Peripheral biomarkers may not be an accurate reflection of what is actually occurring in the CNS [76,229], and the co-occurrence of other disorders in schizophrenia may also interfere with studies on selective biomarkers. Although some very useful animal models of schizophrenia have been developed [8,37,54,95,113,114,230], there remain problems with translation of laboratory animal data to the clinic in schizophrenia [49,113,114,231].

As pointed out by various references in this review, studies on neuroglia and other aspects of schizophrenia in the future should include more comprehensive, well-powered longitudinal studies that consider important factors such as sex, drugs being taken (and the duration of treatment), smoking status, alcohol consumption, subtype differences, nutrition, age of onset, stage of the disorder, brain region differences, methodological aspects, and the prevalence of positive and negative symptoms [59,74,118,153,193,232,233,234,235,236,237,238,239,240,241,242,243]. Where feasible, there should be an application of multiple methodologies, and the possible relationship of the findings to specific symptoms and to patient subtypes (e.g., patients with inflammatory or BBB abnormalities) should be studied [54,59,233,244,245,246,247].

Research to date has suggested some potential drug targets related to glia, but as yet there have not been major breakthroughs, to our knowledge, in antipsychotic development based specifically on studies on their effects on glia. Although variable results have been reported, there have been suggestions that drugs such as antioxidants, anti-inflammatory drugs (including monoclonal antibodies), omega-3 fatty acids, minocycline, drugs acting in epigenetic mechanisms and pre- and probiotics could be useful as adjunctive agents for treating some symptoms of schizophrenia [1,32,36,39,63,234,240,248,249,250,251]. Several naturally occurring compounds of plant or mammalian origin have been proposed as glioprotectives. Quicozes-Santos et al. [60] have listed several of these compounds (resveratrol, curcumin, guanosine, isoflavones, lipoic acid and sulforaphane) and their proposed glioprotective mechanisms. In recent years, a variety of potential new antipsychotics have been tested in clinical trials and show promise; these include drugs acting on muscarinic receptors, the trace amine-associated receptor1 (TAAR1) and 5-HT2A receptors as well as a glycine transporter1 inhibitor, a D-amino acid oxidase inhibitor and a voltage-gated sodium channel blocker (see [3,251]). A new drug targeting muscarinic receptors has recently received FDA approval for the treatment of schizophrenia [252]. In a recent paper, Correll et al. [49] described a number of strategies for the future pharmacotherapy of schizophrenia, including targeting neural networks and circuits, developing biased agonists that provide the selective activation of specific signalling pathways downstream of receptors, and applying molecular polypharmacy to develop drugs that target multiple molecular pathways. Based on our knowledge of the involvement of glia in schizophrenia, it is conceivable that drugs acting on the mechanisms mentioned above in this paragraph could also be having effects on glial function, and it would seem reasonable to monitor such effects on the biomarkers for glia in studies on these drugs since changes in the levels of these biomarkers may be useful in monitoring treatment response over time.

## List of Abbreviations

AMPK	Adenosine monophosphate-activated protein kinase
ATP	Adenosine triphosphate
BBB	Blood–brain barrier
BDNF	Brain-derived neurotrophic factor
cAMP	Cyclic adenosine monophosphate
C1q	Complement component 1q
CNS	Central nervous system
COX2	Cyclooxygenase2
CSF	Cerebrospinal fluid
DISC	Disrupted-in-schizophrenia 1
Erk	Extracellular signal-regulated kinase
FEP	First-episode psychosis
GABA	γ-Aminobutyric acid
GFAP	Glial fibrillary acidic protein
GWAS	Genome-wide association study
hiPSC	Human-induced pluripotent stem cells
HPA Axis	Hypothalamic–pituitary–adrenal axis
5-HT	5-Hydroxytryptamine, Serotonin
INF	Interferon
IL	Interleukin
IP_3_	Inositol 1,4,5-triphosphate
iPSC	Induced pluripotent stem cell
JAK-STAT	Janus kinase-signal transducer and activator of transcription
LPS	Lipopolysaccharide
MAPKS	Mitogen-activated protein kinases
MHC	Major histocompatibility complex
MIA	Maternal immune activation
MK-801	Dizocilpine
MRS	Magnetic resonance spectroscopy
NG2	Neuron-glia antigen 2
NLRP3	Nod-like receptor protein3
NMDA	N-Methyl-D-aspartate
NMDAR	N-Methyl-D-aspartate glutamate receptor
Nrf2	Nuclear factor erythroid 2-related factor2
nVHL	Neonatal ventral hippocampus lesion
OPC	Oligodendrocyte precursor cell
PCP	Phencyclidine
PFC	Prefrontal cortex
PKB	Protein kinase B (also called Akt)
PNS	Peripheral nervous system
RNS	Reactive nitrogen species
ROS	Reactive oxygen species
SCFA	Short-chain fatty acid
SNAP	Synaptic neuron and astrocyte program
SVZ	Subventricular zone
TAAR1	Trace amine-associated receptor1
TGF-β1	Transforming growth factor-β1
TNF	Tumor necrosis factor

## References

[1] Ermakov E.A., Dmitrieva E.M., Parshukova D.A., Kazantseva D.V., Vasilieva A.R., Smirnova L.P. (2021). Oxidative Stress-Related Mechanisms in Schizophrenia Pathogenesis and New Treatment Perspectives. Oxid. Med. Cell. Longev..

[2] Notter T. (2021). Astrocytes in Schizophrenia. Brain Neurosci. Adv..

[3] Tandon R., Nasrallah H., Akbarian S., Carpenter W.T., DeLisi L.E., Gaebel W., Green M.F., Gur R.E., Heckers S., Kane J.M. (2024). The Schizophrenia Syndrome, circa 2024: What We Know and How That Informs Its Nature. Schizophr. Res..

[4] American Psychiatric Association (2022). Diagnostic and Statistical Manual of Mental Disorders.

[5] Correll C.U., Schooler N.R. (2020). Negative Symptoms in Schizophrenia: A Review and Clinical Guide for Recognition, Assessment, and Treatment. Neuropsychiatry Dis. Treat..

[6] Fišar Z. (2023). Biological Hypotheses, Risk Factors, and Biomarkers of Schizophrenia. Prog. Neuropsychopharmacol. Biol. Psychiatry.

[7] Insel T.R. (2010). Rethinking Schizophrenia. Nature.

[8] Dietz A.G., Goldman S.A., Nedergaard M. (2020). Glial Cells in Schizophrenia: A Unified Hypothesis. Lancet Psychiatry.

[9] Murray R.M., Lewis S.W. (1987). Is Schizophrenia a Neurodevelopmental Disorder?. Br. Med. J..

[10] McGrath J.J., Feron F.P., Burne T.H., Mackay-Sim A., Eyles D.W. (2003). The Neurodevelopmental Hypothesis of Schizophrenia: A Review of Recent Developments. Ann. Med..

[11] Ariciloglu F., Ozkartal C.S., Unal G., Dursun S., Cetin M., Muller N. (2017). Neuroinflammation in Schizophrenia: A Critical Review and the Future. Bull. Clin. Psychopharmacol..

[12] Stanca S., Rossetti M., Bokulic Panichi L., Bongioanni P. (2024). The Cellular Dysfunction of the Brain–Blood Barrier from Endothelial Cells to Astrocytes: The Pathway Towards Neurotransmitter Impairment in Schizophrenia. Int. J. Mol. Sci..

[13] Smigielski L., Jagannath V., Rössler W., Walitza S., Grünblatt E. (2020). Epigenetic Mechanisms in Schizophrenia and Other Psychotic Disorders: A Systematic Review of Empirical Human Findings. Mol. Psychiatry.

[14] Li M., Xiao L., Chen X. (2022). Histone Acetylation and Methylation Underlie Oligodendroglial and Myelin Susceptibility in Schizophrenia. Front. Cell. Neurosci..

[15] Zhu B., Ainsworth R.I., Wang Z., Liu Z., Sierra S., Deng C., Callado L.F., Meana J.J., Wang W., Lu C. (2023). Antipsychotic-Induced Epigenomic Reorganization in Frontal Cortex of Individuals with Schizophrenia. eLife.

[16] Owen M.J., O’Donovan M.C., Thapar A., Craddock N. (2011). Neurodevelopmental Hypothesis of Schizophrenia. Br. J. Psychiatry.

[17] McGrath J., Saha S., Chant D., Welham J. (2008). Schizophrenia: A Concise Overview of Incidence, Prevalence, and Mortality. Epidemiol. Rev..

[18] van Rossum J.M. (1966). The Significance of Dopamine-Receptor Blockade for the Mechanism of Action of Neuroleptic Drugs. Arch. Int. Pharmacodyn. Ther..

[19] Seeman P. (1987). Dopamine Receptors and the Dopamine Hypothesis of Schizophrenia. Synapse.

[20] Davis K.L., Kahn R.S., Ko G., Davidson M. (1991). Dopamine in Schizophrenia: A Review and Reconceptualization. Am. J. Psychiatry.

[21] Carlsson M.L., Carlsson A., Nilsson M. (2004). Schizophrenia: From Dopamine to Glutamate and Back. Curr. Med. Chem..

[22] Howes O.D., Kapur S. (2009). The Dopamine Hypothesis of Schizophrenia: Version III—The Final Common Pathway. Schizophr. Bull..

[23] Stahl S.M. (2018). Beyond the Dopamine Hypothesis of Schizophrenia to Three Neural Networks of Psychosis: Dopamine, Serotonin, and Glutamate. CNS Spectr..

[24] Moghaddam B., Javitt D. (2012). From Revolution to Evolution: The Glutamate Hypothesis of Schizophrenia and Its Implication for Treatment. Neuropsychopharmacology.

[25] Mei Y.-Y., Wu D.C., Zhou N. (2018). Astrocytic Regulation of Glutamate Transmission in Schizophrenia. Front. Psychiatry.

[26] Eggers A.E. (2013). A Serotonin Hypothesis of Schizophrenia. Med. Hypotheses.

[27] Hashimoto K., Shimizu E., Iyo M. (2005). Dysfunction of Glia-Neuron Communication in Pathophysiology of Schizophrenia. Curr. Psychiatry Rev..

[28] Cho M., Lee T.Y., Kwak Y.B., Yoon Y.B., Kim M., Kwon J.S. (2019). Adjunctive Use of Anti-Inflammatory Drugs for Schizophrenia: A Meta-Analytic investigation of Randomized Controlled Trials. Aust. N. Z. J. Psychiatry.

[29] Landek-Salgado M.A., Faust T.E., Sawa A. (2016). Molecular Substrates of Schizophrenia: Homeostatic Signaling to Connectivity. Mol. Psychiatry.

[30] Miller B.J., Goldsmith D.R. (2017). Towards an Immunophenotype of Schizophrenia: Progress, Potential Mechanisms, and Future Directions. Neuropsychopharmacology.

[31] Pollak T.A., Drndarski S., Stone J.M., David A.S., McGuire P., Abbott N.J. (2018). The Blood–Brain Barrier in Psychosis. Lancet Psychiatry.

[32] Ouabbou S., He Y., Butler K., Tsuang M. (2020). Inflammation in Mental Disorders: Is the Microbiota the Missing Link?. Neurosci. Bull..

[33] Bernal-Chico A., Tepavcevic V., Manterola A., Utrilla C., Matute C., Mato S. (2023). Endocannabinoid Signaling in Brain Diseases: Emerging Relevance of Glial Cells. Glia.

[34] Ermakov E.A., Melamud M.M., Buneva V.N., Ivanova S.A. (2022). Immune System Abnormalities in Schizophrenia: An Integrative View and Translational Perspectives. Front. Psychiatry.

[35] Murray A.J., Rogers J.C., Katshu M., Liddle P.F., Upthegrove R. (2021). Oxidative Stress and the Pathophysiology and Symptom Profile of Schizophrenia Spectrum Disorders. Front. Psychiatry.

[36] Ansari Z., Pawar S., Seetharaman R. (2022). Neuroinflammation and Oxidative Stress in Schizophrenia: Are These Opportunities for Repurposing?. Postgrad. Med..

[37] de Bartolomeis A., Barone A., Vellucci L., Mazza B., Austin M.C., Iasevoli F., Ciccarelli M. (2022). Linking Inflammation, Aberrant Glutamate-Dopamine Interaction, and Post-Synaptic Changes: Translational Relevance for Schizophrenia and Antipsychotic Treatment: A Systematic Review. Mol. Neurobiol..

[38] Tsamakis K., Galinaki S., Alevyzakis E., Hortis I., Tsiptsios D., Kollintza E., Kympouropoulos S., Triantafyllou K., Smyrnis N., Rizos E. (2022). Gut Microbiome: A Brief Review on Its Role in Schizophrenia and First Episode of Psychosis. Microorganisms.

[39] Xu H., Yang F. (2022). The Interplay of Dopamine Metabolism Abnormalities and Mitochondrial Deficits in the Pathogenesis of Schizophrenia. Transl. Psychiatry.

[40] Neha N.S., Chan A.W., Dursun S.M., Baker G.B. (2024). The Underlying Neurobiological Mechanisms of Psychosis: Focus on Neurotransmission Dysregulation, Neuroinflammation, Oxidative Stress, and Mitochondrial Dysfunction. Antioxidants.

[41] de Oliveira Figueiredo E.C., Cali C., Petrellli F., Bezzi P. (2022). Emerging Evidence for Astrocyte Dysfunction in Schizophrenia. Glia.

[42] Steullet P., Cabungcal J.H., Monin A., Dwir D., O’Donnell P., Cuenod M., Do K.Q. (2016). Redox Dysregulation, Neuroinflammation, and NMDA Receptor Hypofunction: A “Central Hub” in Schizophrenia Pathophysiology?. Schizophr. Res..

[43] de Almeida V., Martins-de-Souza D. (2018). Cannabinoids and Glial Cells: Possible Mechanism to Understand Schizophrenia. Eur. Arch. Psychiatry Clin. Neurosci..

[44] De Picker L., Fransen E., Coppens V., Timmers M., de Boer P., Oberacher H., Fuchs D., Verkerk R., Sabbe B., Morrens M. (2020). Immune and Neuroendocrine Trait and State Markers in Psychotic Illness: Decreased Kynurenines Marking Psychotic Exacerbations. Front. Immunol..

[45] Roberts R.C. (2021). Mitochondrial Dysfunction in Schizophrenia: With a Focus on Postmortem Studies. Mitochondrion.

[46] Yadav M., Kumar N., Kumar A., Jindal D.K., Dahiya M. (2021). Possible Biomarkers and Contributing Factors of Psychosis: A Review. Curr. Pharmacol. Rep..

[47] Zhang Y., Shi H., Yang G., Yang Y., Li W., Song M., Shao M., Su X., Lv L. (2021). Associations Between Expression of Indoleamine 2,3-Dioxygenase Enzyme and Inflammatory Cytokines in Patients with First-Episode Drug-Naïve Schizophrenia. Transl. Psychiatry.

[48] Cuenod M., Steullet P., Cabungcal J.H., Dwir D., Khadimallah I., Klauser P., Conus P., Do K.Q. (2022). Caught in Vicious Circles: A Perspective on Dynamic Feed-Forward Loops Driving Oxidative Stress in Schizophrenia. Mol. Psychiatry.

[49] Correll C.U., Tusconi M., Carta M.G., Dursun S.M. (2024). What Remains to be Discovered in Schizophrenia Therapeutics: Contributions by Advancing the Molecular Mechanisms of Drugs for Psychosis and Schizophrenia. Biomolecules.

[50] Rasband M.N. (2016). Glial Contributions to Neural Function and Disease. Mol. Cell. Proteom..

[51] Wang C., Aleksic B., Ozaki N. (2015). Glia-Related Genes and Their Contribution to Schizophrenia. Psychiatr. Clin. Neurosci..

[52] Timmerman A., Tascio D., Jabs R., Boehlen A., Domingos C., Skubal M., Huang W., Kirchoff F., Heeneberger C., Bilkei-Gorzo A. (2023). Dysfunction of NG2 Glial Cells Affects Neuronal Plasticity and Behavior. Glia.

[53] Lai A.Y., Dhami K.S., Todd K.G. (2009). Moving Past the “Neurocentric” Perspective: A Role for Glia in Neuropsychiatric Disorders. J. Psychiatry Neurosci..

[54] Chang C.-Y., Luo D.-Z., Pei J.-C., Kuo M.-C., Hsieh Y.-C., Lai W.-S. (2021). Not just a Bystander: The Emerging Role of Astrocytes and Research Tools in Studying Cognitive Dysfunctions in Schizophrenia. Int. J. Mol. Sci..

[55] Liu Y., Shen X., Zhang Y., Zheng X., Cepeda C., Wang Y., Duan S., Tong X. (2023). Interactions of Glial Cells with Neuronal Synapses, from Astocytes to Microglia and Oligodendrocyte Lineage Cells. Glia.

[56] Parellada E., Gassó P. (2021). Glutamate and Microglia Activation as a Driver of Dendritic Apoptosis: A Core Pathophysiological Mechanism to Understand Schizophrenia. Transl. Psychiatry.

[57] Brisch R., Wojtylak S., Saniotis A., Steiner J., Gos T., Kumaratilake J., Henneberg M., Wolf R. (2022). The Role of Microglia in Neuropsychiatric Disorders and Suicide. Eur. Arch. Psychiatry Clin. Neurosi..

[58] Hartmann S.M., Heider J., Wust R., Fallgatter A.J., Volkmer H. (2024). Microglia-Neuron Interactions in Schizophrenia. Front. Cell. Neurosci..

[59] Laricchiuta D., Papi M., Decandia D., Panuccio A., Cutuli D., Peciccia M., Mazzeschi C., Petrosini L. (2024). The Role of Glial Cells in Mental Illness: A Systematic Review on Astroglia and Microglia as Potential Players in Schizophrenia and Its Cognitive and Emotional Aspects. Front. Cell. Neurosci..

[60] Quincozes-Santos A., Santos C.L., de Souza Almeida R.R., da Silva A., Thomaz N.K., Fernandes Costa N.L., Becker Weber F., Schmitz I., Scopel Medeiros L., Medeiros L. (2021). Gliotoxicity and Glioprotection: The Dual Role of Glial Cells. Mol. Neurobiol..

[61] Wiseman S. (2024). Glia Moved to the Foreground. Nat. Neurosci..

[62] Beardsley P.M., Hauser K.F. (2014). Glial Modulators as Potential Treatments of Psychostimulant Abuse. Adv. Pharmacol..

[63] Takahashi N., Sakurai T. (2013). Roles of Glia Cells in Schizophrenia: Possible Targets for Therapeutic Approaches. Neurobiol. Dis..

[64] Muller N., Schwarz M.J. (2010). Immune System and Schizophrenia. Curr. Immunol. Rev..

[65] Filman S.G., Cloonan N., Catts V.S., Miller L.C., Wong J., McCrossin T., Cairns M., Weickert C.S. (2013). Increased Inflammatory Markers Identified in the Dorsolateral Prefrontal Cortex of Individuals with Schizophrenia. Mol. Psychiatry.

[66] Khandaker G.M., Meyer U., Jones P.B. (2020). Neuroinflammation and Schizophrenia.

[67] Upthegrove R., Khandaker G.M., Khandaker G.M., Meyer U., Jones P.B. (2020). Cytokines, Oxidative Stress, and Cellular Markers of Inflammation in Schizophrenia. Neuroinflammation and Schizophrenia.

[68] Trepanier M., Hopperton K., Mizrahi R., Mechawar N., Bazinet R. (2016). Postmortem Evidence of Cerebral Inflammation in Schizophrenia: A Systematic Review. Mol. Psychiatry.

[69] Murphy C.E., Walker A.K., Weickert C.S. (2021). Neuroinflammation in Schizophrenia: The Role of Nuclear Factor Kappa B. Transl. Psychiatry.

[70] Mondelli V., Blackman G., Kempton M.J., Pollak T.A., Iyegbe C., Valmaggia L.R., Amminger P., Barrantes-Vidal N., Bressan R., van der Gaag M. (2023). Serum Immune Markers and Transition to Psychosis in Individuals at Clinical High Risk. Brain Behav. Immun..

[71] Van Kesteren C., Gremmels H., De Witte L. (2017). Immune Involvement in the Pathogenesis of Schizophrenia: A Meta-Analysis of Postmortem Brain Studies. Transl. Psychiatry.

[72] Comer A.L., Carrier M., Tremblay M.È., Cruz-Martín A. (2020). The Inflamed Brain in Schizophrenia: The Convergence of Genetic and Environmental Risk Factors That Lead to Uncontrolled Neuroinflammation. Front. Cell. Neurosci..

[73] Tang Y., Tan Y., Palaniyappan L., Yao Y., Luo Q., Li Y. (2024). Epigenetic Profile of the Immune System Associated with Symptom Severity and Treatment Response in Schizophrenia. J. Psychiatr. Neurosci..

[74] Gober R., Dallmeier J., Davis D., Brzostowicki D., de Rivero Vaccari J.P., Cyr B., Barreda A., Sun X., Gultekin S.H., Garamszegi S. (2024). Increased Inflammasome Protein Expression Identified in Microglia from Postmortem Brains with Schizophrenia. J. Neuropathol. Exp. Neurol..

[75] Tusconi M., Dursun S.M. (2024). Editorial: Further Findings in the Role of Inflammation in the Etiology and Treatment of Schizophrenia. Front. Psychiatry.

[76] Warren N., O’Gorman C., Horgan I., Weeratunga M., Halstead S., Moussiopoulou J., Campana M., Yakimov V., Wagner E., Siskind D. (2024). Inflammatory Cerebrospinal Fluid Markers in Schizophrenia Spectrum Disorders: A Systematic Review and Meta-Analysis of 69 Studies With 5710 Participants. Schizophr. Res..

[77] Al-Asmari A.K., Khan M.W. (2014). Inflammation and Schizophrenia: Alterations in Cytokine Levels and Perturbation in Antioxidative Defense Systems. Human. Exp. Toxicol..

[78] Yuan X., Kang Y., Zhuo C., Huang X.F., Song X. (2019). The Gut Microbiota Promotes the Pathogenesis of Schizophrenia via Multiple Pathways. Biochem. Biophys. Res. Commun..

[79] Reale M., Costantini E., Greig N.H. (2021). Cytokine Imbalance in Schizophrenia. From Research to Clinic: Potential Implications for Treatment. Front. Psychiatry.

[80] Vallée A. (2022). Neuroinflammation in Schizophrenia: The Key Role of the WNT/β-Catenin Pathway. Int. J. Mol. Sci..

[81] Kim H., Baek S.-H., Kim J.-W., Ryu S., Lee J.-Y., Kim J.-M., Chung Y.-C., Kim S.-W. (2023). Inflammatory Markers of Symptomatic Remission at 6 Months in Patients with First-Episode Schizophrenia. Schizophrenia.

[82] Trindade P., Nascimento J.M., Casas B.S., Monteverde T., Gasparotto J., Ribeiro C.T., Devalle S., Sauma D., Moreira J.C.F., Gelain D.P. (2023). Induced Pluripotent Stem Cell-Derived Astrocytes from Patients with Schizophrenia Exhibit an Inflammatory Phenotype that Affects Vascularization. Mol. Psychiatry.

[83] Popov P., Chen C., Al-Hakeim H.K., Al-Musawi A.F., Al-Dujaili A.H., Stoyanov D., Maes M. (2024). The Novel Schizophrenia Subgoup “Major Neurocognitive Psychosis” is Validated as a Distinct Class Through the Analysis of Immune-Linked Neurotoxicity Biomarkers and Neurocognitive Deficits. Brain Behav. Immun..

[84] Breitmeyer R., Vogel S., Heider J., Hartmann S.-M., Wust R., Keller A.-L., Binner A., Fitzgerald J.C., Fallgatter A.J., Volkmer H. (2023). Regulation of Synaptic Connectivity in Schizophrenia Spectrum by Mutual Neuron-Microglia Interaction. Commun. Biol..

[85] Patel S., Keating B.A., Dale R.C. (2023). Anti-inflammatory Properties of Commonly Used Psychiatric Drugs. Front. Neurosci..

[86] Palasz E., Wilkaniec A., Stanaszek L., Andrzejewska A., Adamczyk A. (2023). Glia-Neurotrophic Factor Relationships: Possible Role in Pathobiology of Neuroinflammation-Related Brain Disorders. Int. J. Mol. Sci..

[87] Perez-Rodriguez D.R., Blanco-Luguin I., Mendioroz M. (2021). The Participation of Microglia in Neurogenesis: A Review. Brain Sci..

[88] Wohleb E.S. (2016). Neuron-Microglia Interactions in Mental Health Disorders: “For Better, and For Worse”. Front. Immunol..

[89] Monji A., Kato T., Kanba S. (2009). Cytokines and Schizophrenia: Microglia Hypothesis of Schizophrenia. Psychiatry Clin. Neurosci..

[90] Pocock J.M., Kettermann H. (2007). Neurotransmitter receptors on microglia. Trends Neurosci..

[91] Lai A.Y., Todd K.G. (2008). Differential Regulation of Trophic and Proinflammatory Microglial Effectors is Dependent on Severity of Neuronal Injury. Glia.

[92] Zhang X., Wang D., Zhang B., Zhu J., Zhou Z., Cui L. (2020). Regulation of Microglia by Glutamate and its Signal Pathway in Neurodegenerative Diseases. Drug Discov. Today.

[93] Hu J., Baydyuk M., Huang J.K. (2022). Impact of Amino Acids on Microglial Activation and CNS Remyelination. Curr. Opinion Pharmacol..

[94] Bloomfield P.S., Selvaraj S., Veronese M., Rizzo G., Bertoldo A., Owen D.R., Bloomfield M.A.P., Bonoldi I., Kalk N., Turkheimer F. (2016). Microglial Activity in People at Ultra High Risk of Psychosis and in Schizophrenia: An [11C]PBR28 PET Brain Imaging Study. Am. J. Psychiatry.

[95] Barron H., Hafizi S., Andreazza A.C., Mizrahi R. (2017). Neuroinflammation and Oxidative Stress in Psychosis and Psychosis Risk. Int. J. Mol. Sci..

[96] Parkhurst C.N., Yang G., Ninan I., Savas J.N., Yates III J.R., Lafaille J.J., Hempstead B.L., Littman D.R., Gan W.-B. (2013). Microglia Promote Learning-Dependent Synapse Formation Through Brain-Derived Neurotrophic Factor. Cell.

[97] Polyhonen S., Er S., Domansky A., Airavaara M. (2019). Effects of Neurotrophic Factors in Glial Cells in the Central Nervous System: Expression and Properties in Neurodegeneration and Injury. Front. Physiol..

[98] Nieto R.R., Carrasco A., Corral S., Castillo R., Gaspar P.A., Bustamante M.L., Silva H. (2021). BDNF as a Biomarker of Cognition in Schizophrenia/Psychosis: An Updated Review. Front. Psychiatry.

[99] Steiner J., Mawrin C., Ziegeler A., Bielau H., Ullrich O., Bernstein H.-G., Bogerts B. (2006). Distribution of HLA-DR-Positive Microglia in Schizophrenia Reflects Impaired Cerebral Lateralization. Acta Neuropathol..

[100] Goudriaan A., de Leeuw C., Ripke S., Hultman C.M., Sklar P., Sullivan P.F., Smit A.B., Posthuma D., Verheijen M.H.G. (2014). Specific Glial Functions Contribute to Schizophrenia Susceptibility. Schizophr. Bull..

[101] Snijders G.J.L.J., van Zulden W., Sneeboer M.A.M., van Berlekom A.B., van der Geest A.T., Schnieder T., MacIntyre D.J., Hol E.M., Kahn R.S., de Witte L.D. (2021). A Loss of Mature Microglial Markers Without Immune Activation in Schizophrenia. Glia.

[102] Corsi-Zuelli F., Deakin B. (2021). Impaired Regulatory T Cell Control of Astroglial Overdrive and Microglial Pruning in Schizophrenia. Neurosci. Biobehav. Rev..

[103] Murphy C.E., Weickert C.S. (2021). A New Suspect in the Unsolved Case of Neuroinflammation in Schizophrenia. Mol. Psychiatry.

[104] Koskuvi K., Porsti E., Hewitt T., Rasanen N., Wu Y.C., Trontti K., McQuade A., Kalyanaraman S., Ojansuu I., Vaurio O. (2024). Genetic Contribution to Microglial Activation in Schizophrenia. Mol. Psychiatry.

[105] Dalakas M.C., Alexopoulos H., Spaeth P.J. (2020). Complement in Neurological Disorders and Emerging Complement-Targeted Therapeutics. Nat. Rev. Neurol..

[106] Phadke R.A., Brack A., Fournier L.A., Kruzich E., Sha M., Pickard I., Johnson C., Stroumbakis D., Salgado M., Yeung C. (2024). The Schizophrenia Risk Gene C4 Induces Pathological Synaptic Loss by Impairing AMPAR Trafficking. Mol. Psychiatry.

[107] Chen Y., Chu J.M.T., Chang R.C.C., Wong G.T.C. (2022). The Complement System in the Central Nervous System: From Neurodevelopment to Neurodegeration. Biomolecules.

[108] Sekar A., Bialas A.R., de Rivera H., Davis A., Hammond T.R., Kamitaki N., Tooley K., Presumey J., Baum M., van Doren V. (2016). Schizophrenia Risk from Complex Variation of Complement Component 4. Nature.

[109] Mou T.-C.M., Lane M.V., Ireland D.D.C., Verthely D., Tonelli L.H., Clark S.M. (2022). Association of Complement Componenet 4 with Neuroimmune Abnormalities in the Subventriccular Zone in Schizophrenia and Autism Spectrum Disorders. Neurobiol. Dis..

[110] Gubert C., Stertz L., Pfaffenseller B., Panizzutti B.S., Rezin G.T., Massuda R., Streck E.L., Gama C.S., Kapczinski F., Kunz M. (2013). Mitochondrial Activity and Oxidative Stress Markers in Peripheral Blood Mononuclear Cells of Patients with Bipolar Disorder, Schizophrenia, and Healthy Subjects. J. Psychiatr. Res..

[111] Naik E., Dixit V.M. (2011). Mitochondrial Reactive Oxygen Species Drive Proinflammatory Cytokine Production. J. Exp. Med..

[112] Vikhreva O.V., Uranova N.A. (2022). Microglia Reactivity in the Prefrontal Cortex in Schizophrenia with Different Types of Course. Neurosci. Behav. Phys..

[113] Howes O.D., McCutcheon R. (2017). Inflammation and the Diathesis-Stress Hypothesis of Schizophrenia: A Reconceptulization. Transl. Psychiatry.

[114] Mattei D., Schweibold R., Wolf S.A. (2015). Brain in Flames—Animal Models of Psychosis: Utility and Limitations. Neuropsychiatr. Dis. Treat..

[115] Zihni C., Mills C., Matter K., Balda M.S. (2016). Tight Junctions: From Simple Barriers to Multifunctional Molecular Gates. Nat. Rev. Mol. Cell Biol..

[116] Najjar S., Pahlajani S., De Sanctis V., Stern J.N.H., Najjar A., Chong D. (2017). Neurovascular Unit Dysfunction and Blood-Brain Barrier Hyperpermeability Contribute to Schizophrenia Neurobiology; A Theoretical Integration of Clinical and Experimental Evidence. Front. Psychiatry.

[117] Sudo N., Chida Y., Aiba Y., Sonoda J., Oyama N., Yu X.-N., Kubo C., Koga Y. (2004). Postnatal Microbial Colonization Programs the Hypothalamic-Pituitary-Adrenal System for Stress Response in Mice. J. Physiol..

[118] Sharon G., Sampson T.R., Geschwind D.H., Mazmanian S.K. (2016). The Central Nervous System and the Gut Microbiome. Cell.

[119] Kelly J.R., Minuto C., Cryan J.F., Clarke G., Dinan T.G. (2021). The Role of the Gut Microbiome in the Development of Schizophrenia. Schizophr. Res..

[120] Murray N., Ghomi R.H., Nemani K., O’Connor K., Hyland N., Stanton C. (2024). The Influence of Gut Microbiota in Psychosis. The Gut-Brain Axis.

[121] Foster J.A., McVey Neufeld K.A. (2013). Gut-Brain Axis: How the Microbiome Influences Anxiety and Depression. Trends Neurosci..

[122] Hoban A.E., Stilling R.M., Ryan F.J., Shanahan F., Dinan T.G., Claesson M.J., Clarke G., Cryan J.F. (2016). Regulation of Prefrontal Cortex Myelination by the Microbiota. Transl. Psychiatry.

[123] Kraeuter A.K., Phillips R., Sarnyai Z. (2020). The Gut Microbiome in Psychosis from Mice to Men: A Systematic Review of Preclinical and Clinical Studies. Front. Psychiatry.

[124] Zhu F., Guo R., Wang W., Ju Y., Wang Q., Ma Q., Sun Q., Fan Y., Xie Y., Yang Z. (2020). Transplantation of Microbiota from Drug-Free Patients with Schizophrenia Causes Schizophrenia-like Abnormal Behaviors and Dysregulated Kynurenine Metabolism in Mice. Mol. Psychiatry.

[125] Zheng P., Zeng B., Liu M., Chen J., Pan J., Han Y., Liu Y., Cheng K., Zhou C., Wang H. (2019). The Gut Microbiome from Patients with Schizophrenia Modulates the Glutamate-Glutamine-GABA Cycle and Schizophrenia-Relevant Behaviors in Mice. Sci. Adv..

[126] McGuinness A.J., Davis J.A., Dawson S.L., Loughman A., Collier F., O’Hely M., Simpson C.A., Green J., Marx W., Hair C. (2022). A Systematic Review of Gut Microbiota Composition in Observational Studies of Major Depressive Disorder, Bipolar Disorder and Schizophrenia. Mol. Psychiatry.

[127] Nocera A., Nasrallah H.A. (2022). The Association of the Gut Microbiota with Clinical Features in Schizophrenia. Behav. Sci..

[128] Nuncio-Mora L., Lanzagorta N., Nicolini H., Sarmiento E., Ortiz G., Sosa F., Genis-Mendoza A.D. (2023). The Role of the Microbiome in First Episode of Psychosis. Biomedicines.

[129] Misiak B., Łoniewski I., Marlicz W., Frydecka D., Szulc A., Rudzki L., Samochowiec J. (2020). The HPA Axis Dysregulation in Severe Mental Illness: Can We Shift the Blame to Gut Microbiota?. Prog. Neuropsychopharmacol. Biol. Psychiatry.

[130] Erny D., Hrabě de Angelis A.L., Jaitin D., Wieghofer P., Staszewski O., David E., Keren-Shaul H., Mahlakoiv T., Jakobshagen K., Buch T. (2015). Host Microbiota Constantly Control Maturation and Function of Microglia in the CNS. Nat. Neurosci..

[131] Ju S., Shin Y., Han S., Kwon J., Choi T.G., Kang I., Kim S.S. (2023). The Gut-Brain Axis in Schizophrenia: The Implications of the Gut Microbiome and SCFA Production. Nutrients.

[132] Veerkhratsky A., Butt A.M. (2023). Astroglial Functions (Chapter 4). Neuroglia: Function and Pathology.

[133] Schiera G., Di Liegro C.M., Schiro G., Sorbello G., Di Liegro I. (2024). Involvement of Astrocytes in the Formation, Maintenance, and Function of the Blood–Brain Barrier. Cells.

[134] Allen N.J., Eroglu C. (2017). Cell Biology of Astrocyte-Synapse Interactions. Neuron.

[135] Petrelli F., Dallerac G., Pucci L., Cali C., Zehnder T., Sultan S., Lecca S., Chicca A., Ivanov A., Asensio C.S. (2020). Dysfunction of Homeostatic Control of Dopamine by Astrocytes in the Developing Prefrontal Cortex Leads to Cognitive Impairments. Mol. Psychiatry.

[136] Zehnder T., Petrelli F., Romanos J., de Oliveira Figueiredo E.C., Lewis T.L., Deglon N., Polleux F., Santello M., Bezzi P. (2021). Mitochondrial Biogenesis in Developing Astrocytes Regulates Astrocyte Maturation and Synapse Formation. Cell Rep..

[137] Hughes E.G., Elmariah S.B., Balice-Gordon R.J. (2010). Astrocyte Secreted Proteins Selectively Increase Hippocampal GABAergic Axon Length, Branching and Synaptogenesis. Mol. Cell. Neurosci..

[138] Leferink P.S., Heine V.M. (2018). The Healthy and Diseased Microenvironments Regulate Oligodendrocyte Properties: Implications for Regenerative Medicine. Am. J. Pathol..

[139] Miyamoto N., Maki T., Shindo A., Liang A.C., Maeda M., Egawa N., Itoh K., Lo E.K., Lok J., Ihara M. (2015). Astrocytes Promote Oligodendrogenesis after White Matter Damage via Brain-Derived Neurotrophic Factor. J. Neurosci..

[140] Ling E., Nemesh J., Goldman M., Kamitaki N., Reed N., Handsaker R.E., Genovese G., Vogelgsang J.S., Gerges S., Kashin S. (2024). A Concerted Neuron-Astrocyte Program Declines in Ageing and Schizophrenia. Nature.

[141] Oliveira J.P., Araque A. (2022). Astrocyte Regulation of Neural Circuit Activity and Network States. Glia.

[142] Chung W.-S., Allen N.J., Eroglu C. (2015). Astrocytes Control Synapse Formation, Function, and Elimination. Cold Spring Harb. Perspect. Biol..

[143] Hu W., MacDonald M.I., Elswick D.E., Sweet R.A. (2015). The Glutamate Hypothesis of Schizophrenia: Evidence from Human Brain Tissue Studies. Ann. N. Y. Acad. Sci..

[144] Gentile M.T., D’Amato L.C. (2018). Astrocyte Physiology and Pathology.

[145] Fiebig C., Keiner S., Ebert B., Schaffner I., Jagasia R., Lie D.C., Beckervordersandforth R. (2019). Mitochondrial Dysfunction in Astrocytes Impairs the Generation of Reactive Astrocytes and Enhances Cell Death in the Cortex Upon Phtothrombotic Lesion. Front. Mol. Neurosci..

[146] Chen Y., Qin C., Huang J., Tang X., Liu C., Huang K., Xu J., Guo G., Tong A., Zhou L. (2020). The Role of Astrocytes in Oxidative Stress of Central Nervous System: A Mixed Blessing. Cell Prolif..

[147] Gouix E., Buisson A., Nieoullon A., Kerkerian-Le Goff L., Tauskela J.S., Biondeau N., Had-Aissouni L. (2014). Oxygen Glucose Deprivation-Induced Astrocyte Dysfunction Provokes Neuronal Death Through Oxidtive Stress. Pharmacol. Res..

[148] Rothstein J.D., Dykes-Hoberg M., Pardo C.A., Bristol L.A., Jin L., Kunci R.W., Kanai Y., Hediger M.A., Wang Y., Schielke J.P. (1996). Knockout of Glutamate Transporters Reveals a Major Role for Astroglial Transport in Excitotoxicity and Clearance of Glutamate. Neuron.

[149] Purushotham S.S., Buskila Y. (2023). Astrocytic Modulation of Neuronal Signalling. Front. Netw. Physiol..

[150] Sonnewald U., Schousboe A. (2016). Introduction to the Glutamate–Glutamine Cycle. Adv. Neurobiol..

[151] Hashimoto K., Engberg G., Shimizu E., Nordin C., Lindström L.H., Iyo M. (2005). Elevated Glutamine/Glutamate Ratio in Cerebrospinal Fluid of First Episode and Drug Naive Schizophrenic Patients. BMC Psychiatry.

[152] Pavăl D., Gherghel-Pavăl N., Căpățînă O.O., Stan A., Micluția I.V., Giné-Servén E. (2023). The Importance of Cerebrospinal Fluid Investigation in First-Episode Psychosis. Yale J. Biol. Med..

[153] Bernstein H.-G., Dobrowolny H., Keilhoff G., Bogerts B., Steiner J. (2018). Reduced Density of DISC1 Expressing Astrocytes in the Dentate Gyrus but Not in the Subventricular Zone in Schizophrenia. Neuropsychopharmacology.

[154] Labrie V., Wong A.H.C., Roder J.C. (2012). Contributions of the D-Serine Pathway to Schizophrenia. Neuropharmacology.

[155] Nishikawa T., Umino A., Umino M., Riederer P., Laux G., Nagatsu T., Le W., Riederer C. (2022). D-Serine in the Treatment of Psychosis. NeuroPsychopharmacotherapy.

[156] Stone T.W. (1993). Neuropharmacology of Quinolinic and Kynurenic Acids. Pharmacol. Rev..

[157] Kessler M., Terramani T., Lynch G., Baudry M. (1989). A Glycine Site Associated with N-Methyl D-Aspartic Acid Receptors: Charcterization and Identification of a New Class of Antagonists. J. Neurochem..

[158] Sapienza J., Spangaro M., Guillemin G.J., Comai S., Bosia M. (2023). Importance of the Dysregulation of the Kynurenine pathway on Cognition in Schizophrenia: A Systematic Review of Clinical Studies. Eur. Arch. Psychiatry Clin. Neurosi..

[159] Papouin T., Henneberger C., Rusakov D.A., Oliet S.H.R. (2017). Astroglial Versus Neuronal D-Serine: Fact Checking. Trends Neurosci..

[160] Wolosker H., Balu D.T., Coyle J.T. (2016). The Rise and Fall of the D-Serine-Mediated Gliotransmission Hypothesis. Trends Neurosci..

[161] Wolosker H., Balu D.T. (2020). D-Serine as the Gatekeeper of NMDA Receptor Activity: Implications for the Pharmacologic Management of Anxiety Disorders. Transl. Psychiatry.

[162] Krishnan K.S., Billups B. (2023). ASC Transporters Mediate D-serine Transport into Astrocytes Adjacent to Synapses in the Mouse Brain. Biomolecules.

[163] Plitman E., Iwata Y., Caravaggio F., Nakajima S., Chung J.K., Gerretsen P., Kim J., Takeuchi H., Chakavarty M.M., Remington G. (2017). Kynurenic Acid in Schizophrenia: A Systematic Review and Meta-Analysis. Schizophr. Bull..

[164] Hilmas C., Pereira E.F., Alkondon M., Rassoulpour A., Schwarcz R., Albuquerque E.X. (2001). The Brain Metabolite Kynurenic Acid Inhibits Alpha7 Nicotinic Receptor Activity and Increases Non-Alpha7 Nicotinic Receptor Expression: Physiopathological Implications. J. Neurosci..

[165] Dobelis P., Staley K.J., Cooper D.C. (2012). Lack of Modulation of Nicotinic Acetylcholine Alpha-7 Receptor Currents by Kynurenic Acid in Adult Hippocampal Interneurons. PLoS ONE.

[166] Albuquerque E.X., Schwarcz R. (2013). Kynurenic Acid as an Antagonist of α7 Nicotinic Acetylcholine Receptors in the Brain: Facts and Challenges. Biochem. Pharmacol..

[167] Schwarcz R., Stone T.W. (2017). The Kynurenine Pathway and the Brain: Challenges, Controversies and Promises. Neuropharmacology.

[168] Bai M.Y., Lovejoy D.B., Guillemin G.J., Kozak R., Stone T.W., Koola M.M. (2021). Galantamine-Memantine Combination and Kynurenine Pathway Enzyme Inhibitors in the Treatment of Neuropsychiatric Disorders. Complex. Psychiatry.

[169] Chen J., Zou J., Huang P., Gao X., Lun J., Li Y., Gong Z., Cao H. (2024). KYNA Ameliorates Glutamate Toxicity of HAND by Enhancing Glutamate Uptake in A2 Astrocytes. Int. J. Mol. Sci..

[170] Erhardt S., Schwieler L., Nilsson L., Linderholm K., Engberg G. (2007). The Kynurenic Acid Hypothesis of Schizophrenia. Physiol. Behav..

[171] Sapienza J., Agostoni G., Dall’Acqua S., Sut S., Nasini S., Martini F., Marchesi A., Bechi M., Buonocore M., Cocchi F. (2024). The Kynurenine Pathway in Treatment-Resistant Schizophrenia at the Crossroads Between Pathophysiology and Pharmacotherapy. Schizophr. Res..

[172] Hatzimanolis A., Foteli S., Xenaki L.-A., Selakovic M., Dimitrakopoulos S., Vlachos I., Kosteletos I., Soldatos R.-F., Gazouli M., Chatzipanagiotou S. (2024). Elevated Serum Kynurenic Acid in Individuals with First-Episode Psychosis and insufficient Response to Antipsychotics. Schizophrenia.

[173] Karpinski P., Samochowiez J., Sasiadek M.M., Laczmanski L., Misiak B. (2020). Analysis of Global Gene Expression at Seven Brain Regions of Patients with Schizophrenia. Schizophr. Res..

[174] De Keyser J., Mostert J.P., Koch M.W. (2008). Dysfunctional Astrocytes as Key Players in the Pathogenesis of Central Nervous System Disorders. J. Neurol. Sci..

[175] Pinjari O.F., Dasgupta S.K., Okusaga O.O. (2022). Plasma Soluble P-Selectin, Interleukin-6 and S100B Protein in Patients with Schizophrenia: A Pilot Study. Psychiatr. Q..

[176] Jeon P., Mackinley M., Theberge J., Palaniyapan I. (2021). The Trajectory of Putative Astroglial Dysfunction in First Episode Schizophrenia: A Longitudinal 7-Tesla MRS Study. Sci. Rep..

[177] Daverey A., Agrawal S.K. (2016). Curcumin Alleviates Oxidative Stress and Mitochondrial Dysfunction in Astrocytes. Neuroscience.

[178] Steiner J., Schiltz K., Walter M., Wunderlich M.T., Keilhoff G., Brisch R., Bielau H., Bernstein H.-G., Bogerts B., Schroeter M.L. (2010). S100B Serum Levels Are Closely Correlated with Body Mass Index: An Important Caveat in Neuropsychiatric Research. Psychoneuroendocrinology.

[179] Aleksovska K., Leoncini E., Bonassi S., Cesario A., Boccia S., Frustaci A. (2014). Systematic Review and Meta-Analysis of Circulating S100B Blood Levels in Schizophrenia. PLoS ONE.

[180] Schümberg K., Polyakova M., Steiner J., Schroeter M.L. (2016). Serum S100B Is Related to Illness Duration and Clinical Symptoms in Schizophrenia—A Meta-Regression Analysis. Front. Cell. Neurosci..

[181] Michinaga S., Koyama Y. (2019). Dual Roles of Astrocyte-Derived Factors in Regulation of Blood-Brain Barrier Function after Brain Damage. Int. J. Mol. Sci..

[182] Madsen P.M., Desu H.L., de Rivero Vaccari J.P., Florimon Y., Ellman D.G., Keane R.W., Clausen B.H., Lambertsen K.L., Brambilla R. (2020). Oligodendrocytes Modulate the Immune-Inflammatory Response in EAE via TNFR2 Signaling. Brain Behav. Immun..

[183] Boccazzi M., Raffaele S., Fumagalli M. (2022). Not Only Myelination: The Immune-Inflammatory Functions of Oligodendrocytes. Neural. Regen. Res..

[184] Vanes L.D., Mouchlianitis E., Barry E., Patel K., Wong K., Shergill S.S. (2019). Cognitive Correlates of Abnormal Myelination in Psychosis. Sci. Rep..

[185] Giotakos O. (2019). Is Psychosis a Dysmyelination-Related Information-Processing Disorder?. Psychiatriki.

[186] Zhang R., He J., Zhu S., Zhang H., Wang H., Adilijiang A., Kong L., Wang J., Kong J., Tan Q. (2012). Myelination Deficit in a Phencyclidine-Induced Neurodevelopmental Model of Schizophrenia. Brain Res..

[187] Hof P.R., Haroutunian V., Frierich V.I., Byne W., Buition C., Perl D.P., Davis K.L. (2003). Loss and Altered Space Distribution of Oligodendrocytes in the Superior Frontal Gyrus in Schizophrenia. Biol. Psychiatry.

[188] Mighdoll M.I., Tao R., Kleinman J.E., Hyde T.M. (2015). Myelin, Myelin-Related Disorders, and Psychosis. Schizophr. Res..

[189] Uranova N.A., Vikhreva O.V., Rachmanova V.I., Orlovskaya D.D. (2011). Ultrastructural Alterations of Myelinated Fibers and Oligodendrocytes in the Prefrontal Cortex in Schizophrenia: A Postmortem Morphometric Study. Schizophr. Res. Treat..

[190] Maas D.A., Valles A., Martens G.J.M. (2017). Oxidative Stress, Prefrontal Cortex Hypomyelination and Cognitive Symptoms in Schizophrenia. Transl. Psychiatry..

[191] Windrem M.S., Osipovitch M., Liu Z., Bates J., Chandler-Militello D., Zou L., Munir J., Schanz S., McCoy K., Miller R.H. (2017). Human iPSC Glia Mouse Chimeras Reveal Glial Contributions to Schizophrenia. Cell Stem Cell.

[192] Domingues H.S., Portugal C.C., Socodato R., Relvas J.B. (2016). Oligodendrocyte, Astrocyte, and Microglia Crosstalk in Myelin Development, Damage, and Repair. Front. Cell Dev. Biol..

[193] Sun M., You H., Hu X., Luo Y., Zhang Z., Song Y., An J., Lu H. (2023). Microglia-Astrocyte Interaction in Neural Development and Neural Pathogenesis. Cells.

[194] Liddelow S.A., Guttenplan K.A., Clarke L.E., Bennett F.C., Bohlen C.J., Schirmer L., Bennett M.L., Munch A.E., Chung W.S., Peterson T.C. (2017). Neurotoxic Reactive Astrocytes are Induced by Activated Microglia. Nature.

[195] Merril J.E., Ignarro L.J., Sherman M.P., Melinek J., Lane T.E. (1993). Microglial Cell Cytotoxicity of Oligodendrocytes is Mediated Through Nitric Oxide. J. Immunol..

[196] Buntinx M., Moreels M., Vandenabeele F., Lambrichts I., Raus J., Steels P., Stinissen P., Ammeloot M. (2004). Cytokine-Induced Cell Death in Human Oligodendroglial Cell Lines: I. Synergistic Effects of IFN-gamma and TNF-alpha on Apoptosis. J. Neurosci. Res..

[197] Li J., Baud O., Vartanian T., Volpe J.J., Rosenberg P.A. (2005). Peroxynitrite Generated by Inducible Nitric Oxide Synthase and NADPH Oxidase Mediates Microglial Toxicity to Oligodendrocytes. Proc. Natl. Acad. Sci. USA.

[198] Uranova N.A., Vikhreva O.V., Rakhmanova V.I., Orlovskaya D.D. (2020). Dystrophy of Oligodendrocytes and Adjacent Microglia in Prefrontal Gray Matter in Schizophrenia. Front. Psychiatry..

[199] Konopaske G.T., Dorph-Petersen K.-A., Sweet R.A., Pieri J.N., Zhang W., Sampson A.R., Lewis D.A. (2008). Effect of Chronic Antipsychotic Exposure on Astrocyte and Oligodendrocyte Numbers in Macaque Monkeys. Biol. Psychiatry.

[200] Kato T., Monji A., Hashioka S., Kanba S. (2007). Risperidone Significantly Inhibits Interferon-gamma-Induced Microglial Activation in vitro. Schizophr. Res..

[201] MacDowell K.S., Garcia-Bueno B., Madrigal J.L., Parellada M., Arango C., Mico J.A., Leza J.C. (2013). Risperidone Normalizes Increased Inflammatory Parameters and Restores Anti-Inflammatory Pathways in a Model of Neuroinflammation. Int. J. Neuropsychopharmacol..

[202] Bian Q., Kato T., Monji A., Hashioka S., Mizoguchi Y., Horikawa H., Kanba S. (2008). The Effect of Atypical Antipsychotics, Perospirone, Ziprasidone and Quetiapine on Microglial Ativation Induced by Interferon-gamma. Progr. Neuropsychopharmacol. Biol. Psychiatry.

[203] Zheng L.T., Hwang J., Ock J., Lee M.G., Lee W.-H., Suk K. (2008). The Antipsychotic Spiperone Attenuates Inflammatory Response in Cultured Microglia via the Reduction of Proinflammatory Cytokine Expression and Nitric Oxide Production. J. Neurochem..

[204] Kato T., Mizoguchi Y., Monji A., Horikawa H., Suzuki S.O., Seki Y., Iwaki T., Hashioka S., Kanba S. (2008). Inhibitory effects of aripiprazole on interferon-gamma-induced microglial activation via intracellular^2+^ regulation in vitro. J. Neurochem..

[205] Long Y., Wang Y., Shen Y., Huang J., Li Y., Wu R., Zhao J. (2023). Minocycline and Antipsychotics Inhibit Inflammatory Responses in BV-2 Microglia Activated by LPS via Regulating the MAPKs/JAK-STAT Signaling Pathway. BMC Psychiatry.

[206] Bongarzone E.R., Howard S.G., Schonmann V., Campagnoni A.T. (1998). Identification of the Dopamine D3 Receptor in Oligodendrocyte Precursors: Potential Role in Regulating Differentiation and Myelin Formation. J. Neurosci..

[207] Akkouh I.A., Hribkova H., Grabiec M., Budinska E., Szabo A., Kasparek T., Andreassen O.A., Sun Y.-M., Djurovic S. (2022). Derivation and Molecular Characterization of Morphological Subpopulation of Human iPSC Astrocytes Reveal a Potential Role in Schizophrenia and Clozapine Response. Schizophr. Bull..

[208] Torres-Carmona E., Nakajima S., Iwata Y., Ueno F., Stefan C., Song J., Abdolizadeh A., Koizumi M.T., Kambari Y., Amaev A. (2024). Clozapine Treatment and Astrocyte Activity in Treatment Resistant Schizophrenia: A Proton Magnetic Resonance Spectroscopy Study. Schizophr. Res..

[209] Yuhas Y., Ashkenazi S., Berernt E., Weizman A. (2022). Clozapine Suppresses the Gene Expression and the Production of Cytokines and Up-Regulates Cyclooxygenase 2 mRNA in Human Astroglial Cells. Brain Sci..

[210] Vallejo-Illarramendi A., Torres-Ramos M., Melone M., Conti F., Matute C. (2005). Clozapine Reduces GLT-1 Expression and Glutamate Uptake in Astrocyte Cultures. Glia.

[211] Tanahashi S., Yamamura S., Nakagawa M., Motomura E., Okada M. (2012). Clozapine, but not Haloperidol, Enhances Glial D-Serine and L-Glutamate Release in Rat Frontal Cortex and Primary Cultured Astrocytes. Br. J. Pharmacol..

[212] Okada M., Fukuyama K., Motomura E. (2022). Dose-Dependent Biphasic Action of Quetiapine on AMPK Signalling via 5-HT7 Receptor: Exploring Pathophysiology of Clinical and Adverse Effects of Quetiapine. Int. J. Mol. Sci..

[213] Yu K., Zhou H., Chen Z., Lei Y., Wu J., Yuan Q., He J. (2024). Mechanism of cognitive impairment and white matter damage in the MK-801 mice model of schizophrenia treated with quetiapine. Behav. Brain Res..

[214] Chedrawe M.A.J., Holman S.P., Lamport A.-C., Akay T., Robertson G.S. (2018). Pioglitazone is Superior to Quetiapine, Clozapine and Tamoxifen at Alleviating Experimental Autoimmune Encephalomyelitis in Mice. J. Neuroimmunol..

[215] Apam-Castillejos D.J., Tendilla-Beltran H., Vazquez-Roque R.A., Vazquez-Hernandez A.J., Fuentes-Melel E., Garcia-Dolores F., Diaz A., Flores G. (2022). Second-Generation Antipsychotic Olanzapine Attenuates Behavioral and Prefrontal Cortex Synaptic Plasticity Deficits in a Neurodevelopmental Schizophrenia-Related Rat Model. J. Chem. Neuroanat..

[216] Kimoto S., Okuda A., Toritsuka M., Yamauchi T., Makinodan M., Okuda H., Tatsumi K., Nakamura Y., Wanaka A., Kishimoto T. (2011). Olanzapine Stimulates Proliferation but Inhibits Differentiation in Rat Oligodendrocyte Precursor Cell Cultures. Progr. Neuropsychopharmacol. Biol. Psychiatry.

[217] Saibro-Girardi C., Scheibel I.M., Santos L., Bittencourt R.R., Frohlich N.T., dos Reis Possa L., Moreira J.C.F., Gelain D.P. (2023). Bexarotene Drives the Self-Renewing Proliferation of Adult Neuronal Stem Cells, Promotes Neuron-Glial Fate Shift, and Regulates Late Neuronal Differentiation. J. Neurochem..

[218] He J., Huang Y., Liu H., Sun X., Wu J., Zhang Z., Liu L., Zhou C., Jiang S., Huang Z. (2020). Bexarotene Promotes Microglia/Macrophages—Specific Brain-Derived Neurotrophic Factor Expression and Axon Sprouting After Traumatic Brain Injury. Exp. Neurol..

[219] Lerner V., Miodownik C., Gibel A., Kovalyonok E., Shleifer T., Goodman A.B., Ritsner M.S. (2008). Bexarotene s Add-On to Antipsychotic Treatment in Schizophrenia Patients: A Pilot Open-Label Trial. Clin. Neuropharmacol..

[220] Lerner V., Miodownik C., Gibel A., Sirota P., Bush I., Elliot H., Benatov R., Ritsner M.S. (2013). The Retinoid X Receptor Agonist Bexarotene Relieves Positive Symptoms of Schizophrenia: A 6-Week, Randomized, Double-Blind, Placebo-Controlled Multicenter Trial. J. Clin. Psychiatry.

[221] Schmitz I., da Silva A., Bobermine L.D., Goncaives C.-A., Steiner J., Quincozes-Santos A. (2023). The Janus Face of Antipsychotics in Glial Cells: Focus on Glioprotection. Exp. Biol. Med..

[222] He M., Fan J., Zhou R., Gao G., Li R., Zuo Y.F., Li B., Li Y., Sun T. (2022). NLRP3/Caspase-1-Mediated Pyroptosis of Astrocytes Induced by Antipsychotics is Inhibited by a Histamine H1 Receptor-Selective Agonist. Front. Aging Neurosci..

[223] Skene N.G., Bryois J., Bakken T.E., Breen G., Crowley J.J., Gaspar H.A., Giusti-Rodriguez P., Hodge R.D., Miller J.A., Munoz-Manchado A.B. (2018). Genetic Identification of Brain Cell Types Underlying Schizophrenia. Nat. Genet..

[224] Siletti K., Hodge R., Albach A.M., Lee K.W., Ding S.-L., Hu L., Lonnerberg P., Bakken T., Casper T., Clark M. (2023). Transcriptomic Diversity of Cell Types Across the Adult Human Brain. Science.

[225] Wu Y., Zhang C.-Y., Wang L., Li Y., Xiao X. (2023). Genetic Insights of Schizophrenia via Single Cell RNA-Sequencing Analyses. Schizophr. Bull..

[226] Ruzicka W.B., Mohammadi S., Fullard J.F., Davila-Veelderrain J., Subburaju S., Tso D.R., Hourihan M., Jiang S., Lee H.-C., Bendl J. (2024). Single-Cell Multi-Cohort Dissection of the Schizophrenia Transcriptome. Science.

[227] Thrupp N., Frigerio C.S., Wolfs L., Skene N.G., Fattorelli N., Poovathingal S., Fourne Y., Matthews P.M., Theys T., Mancuso R. (2020). Single-Nucleus RNA-Seq is Not Suitable for Detection of Microglial Activation Genes in Humans. Cell Rep..

[228] Minichino A., Brondino N., Solmi M., Del Giovane C., Fusar-Poli P., Burnet P., Cipriani A., Lennox B.R. (2021). The Gut-Microbiome as a Target for the Treatment of Schizophrenia: A Systematic Review and Meta-Analysis of Randomised Controlled Trials of Add-on Strategies. Schizophr. Res..

[229] Goff D.C., Romero K., Paul J., Mercedes Perez-Rodriguez M., Crandall D., Potkin S.G. (2016). Biomarkers for Development in Early Psychosis: Current Issues and Promising Directions. Eur. Neuropsychopharmacol..

[230] Winship I.R., Dursun S.M., Baker G.B., Balista P.A., Kandratavicius L., Maia-de-Oliveira J.P., Hallak J., Howland J.G. (2019). An Overview of Animal Models Related to Schizophrenia. Can. J. Psychiatry.

[231] Kesby J.P., Eyles D.W., McGrath J.J., Scott J.G. (2018). Dopamine, Psychosis and Schizophrenia: The Widening Gap Between Basic and Clinical Neuroscience. Transl. Psychiatry.

[232] Vindegaard N., Speyer H., Nordentoft M., Rasmussen S., Eriksen Benros M. (2021). Gut Microbial Changes of Patients with Psychotic and Affective Disorders: A Systematic Review. Schizophr. Res..

[233] Hoang D., Xu Y., Lutz O., Bannai D., Zeng V., Bishop J.R., Keshavan M., Lizanao P. (2022). Inflammatory Subtypes in Antipsychotic-Naïve First-Episode Schizophrenia are Associated with Altered Brain Morphology and Topologial Organization. Brain Behav. Immun..

[234] Zhou K., Baranova A., Cao H., Sun J., Zhang F. (2024). Gut Microbiome and Schizophrenia: Insights from Two-Sample Mendelian Randomization. Schizophrenia.

[235] Woodberry K.A., Shapiro D.I., Bryant C., Seidman L.J. (2016). Progress and Future Directions in Research on the Psychosis Prodrome. Harv. Rev. Psychiatry.

[236] Potkin S.G., Kane J.M., Correll C.U., Lindenmayer J.-P., Agid O., Marder S.R., Olfson M., Howes O.D. (2020). The Neurobiology of Treatment-Resistant Schizophrenia: Paths to Antipsychotic Resistance and a Roadmap for Future Research. NPJ Schizophr..

[237] Khandaker G.M., Cousins L., Deakin J., Lennox B.R., Yolken R., Jones P.B. (2015). Inflammation and Immunity in Schizophrenia: Implications for pathophysiology and Treatment. Lancet Psychiatry.

[238] Gogos A., van den Buuse M. (2023). Sex Differences in Psychosis; Focus on Animal Models. Curr. Topics Behav. Neurosci..

[239] Breach M.R., Lenz K.M. (2023). Sex Differences in Neurodevelopmental Disorders: A Key Role for the Immune System. Curr. Top. Behav. Neurosci..

[240] Salehi M.A., Zafari R., Mohammadi S., Farahani M.S., Dolatshahi M., Harandi H., Poopak A., Dager S.R. (2024). Brain-Based Sex Differences in Schizophrenia: A Systematic Review of fMRI Studies. Hum. Brain Map..

[241] Samizadeh M.A., Abdollahi-Keyvani S.T., Fallah H., Beigi B., Motamedi-Manesh A., Adibian S., Vaseghi S. (2024). Sex Difference Alters the Behavioral and Cognitive Performance in a Rat Model of Schizophrenia Induced by Sub-Chronic Ketamine. J. Psychiatr. Res..

[242] Schneider E., O’Riordan K.J., Clarke G., Cryan J.F. (2024). Feeding Gut Microbes to Nourish the Brain: Unravelling the Diet-Microbiota-Gut-Brain Axis. Nat. Metab..

[243] Fond G., d’Albis M.-A., Jamain S., Tamouza R., Arango C., Fleischhacker W.W., Glenthoj B., Lewke M., Lewis S., McGuire P. (2015). The Promise of Biological Markers for Treatment Response in First-Episode Psychosis: A Systematic Review. Schizophr. Bull..

[244] Wawrzczak-Bargieła A., Bilecki W., Maćkowiak M. (2023). Epigenetic Targets in Schizophrenia Development and Therapy. Brain Sci..

[245] Yue W., Huang H., Duan J. (2022). Potential Diagnostic Biomarkers for Schizophrenia. Med. Rev..

[246] Chen E.Y.H., Wong S.M.Y. (2024). Unique Challenges in Biomarkers for Psychotic Disorders. Brain Sci..

[247] Seeman M.V. (2023). What is the Significance of the Impact of Antipsychotics on the Gut Microbiome?. Expert Opin. Drug Metab. Toxicol..

[248] Pandurangi A.K., Buckley P.F., Khandaker G.M., Meyer U., Jones P.B. (2019). Inflammation, Antipsychotic Drugs, and Evidence for Effectiveness of Anti-Inflammatory Agents in Schizophrenia. Inflammation and Schizophrenia.

[249] Hansen N., Malchow B. (2023). Monoclonal Antibody Therapy in Autoantibody-Associated Psychotic Disorders and Schizophrenia: Narrative Reviews of Past and Current Clinical Trials. Psychiatr. Danub..

[250] Weickert T.W., Jacomb I., Lenroot R., Lappin J., Weinberg D., Brooks W.S., Brown D., Pellen D., Kindler J., Mohan A. (2024). Adjunctive Canakinumab Reduces Peripheral Inflammation Markers and Improves Positive Symptoms in People with Schizophrenia and Inflammation: A Randomized Control Trial. Brain Behav. Immun..

[251] Kantrowitz J.T., Correll C.U., Jain R., Cutler A.J. (2023). New Developments in the Treatment of Schizophrenia: An Expert Roundtable. Int. J. Neuropsychopharmacol..

[252] Dolgin E. (2024). Revolutionary Drug for Schizophrenia Receives US Approval. Nature.

